# Challenges and Progress in Nonsteroidal Anti-Inflammatory Drugs Co-Crystal Development

**DOI:** 10.3390/molecules26144185

**Published:** 2021-07-09

**Authors:** Ilma Nugrahani, Rismaya Desti Parwati

**Affiliations:** Department of Pharmacochemistry, School of Pharmacy, Bandung Institute of Technology, Bandung 40132, Indonesia; rismayadesti@gmail.com

**Keywords:** challenges, progress, multicompoent, co-crystal, salt co-crystal, NSAID

## Abstract

Co-crystal innovation is an opportunity in drug development for both scientists and industry. In line with the “green pharmacy” concept for obtaining safer methods and advanced pharmaceutical products, co-crystallization is one of the most promising approaches to find novel patent drugs, including non-steroidal anti-inflammatory drugs (NSAID). This kind of multi-component system improves previously poor physicochemical and mechanical properties through non-covalent interactions. Practically, there are many challenges to find commercially viable co-crystal drugs. The difficulty in selecting co-formers becomes the primary problem, followed by unexpected results, such as decreased solubility and dissolution, spring and parachute effect, microenvironment pH effects, changes in instability, and polymorphisms, which can occur during the co-crystal development. However, over time, NSAID co-crystals have been continuously updated regarding co-formers selection and methods development.

## 1. Introduction

Non-steroidal anti-inflammatory drugs (NSAID) are antipyretic, anti-inflammatory, and analgesic agents commonly used to treat muscle pain, dysmenorrhea, rheumatism, pyrexia, gout, migraine, etc. These drugs also are combined with opioids to treat acute trauma cases [1]. The use of NSAID is increasing every year. In 2018, NSAID prescriptions increased by 40.7%, the most significant percentage prescribed to women on long-term treatment. Paracetamol and ibuprofen are the most frequently prescribed NSAID for chronic therapy using fixed-dose combination treatment [2,3,4].

Based on their chemical structure and selectivity, NSAID are divided into salicylic acid derivatives (aspirin, ethenzamide, diflunisal), para-aminophenol derivatives (paracetamol), indoles (etodolac, indomethacin), hetero-acyl acetic acid (diclofenac, ethyl diclofenac, ketorolac), aryl-propionic acid derivatives (ketoprofen, naproxen, ibuprofen, flurbiprofen), anthralinic acid derivatives (mefenamic acid, flufenamic acid, nifluminic acid, meclo-fenamic acid), enolic acid derivatives (tenoxicam, piroxicam, meloxicam), and diaryl-heterocycles or selective cyclooxygenase (COX)-2 inhibitors (celecoxib, etoricoxib) [1].

NSAID are classified in the Biopharmaceutics Classification System (BCS) as class II, because they have low solubility and high permeability [5,6]. The low solubility of NSAID can cause many problems in using and producing these drugs, especially regarding the onset of action. NSAID are the most widely used drugs for pain relief, so a fast onset of action is needed [6]. It is a challenge to develop an optimal potential effect. Various attempts have been made to increase the solubility, such as the formation of salts, polymorphs, solvates, and hydrates [7]. Nowadays, most NSAID on the market are in their salt form, such as diclofenac sodium, diclofenac potassium, ibuprofen, etc. However, salt formation can only be applied to active pharmaceutical ingredients (APIs), forming ions. It is a problem for some compounds that are difficult to ionize, such as zaltoprofen (ZFN). ZFN is included in the propionic acid group and is an effective NSAID to treat acute somatic pain [8], chronic inflammation, rheumatoid arthritis, and phase II clinical trials for giant tenosynovial tumor cells therapy [9]. Polymorphs are not preferred because of the danger of polymorphic transformation, which affects the formulated product [10]. Therefore, it is necessary to develop new approaches to modify or improve the physicochemical properties of these drugs [11,12].

The Food and Drug Administration (FDA) defines co-crystals as crystalline materials consisting of two or more different molecules. Usually, APIs and co-crystal formers (co-formers) form a crystal lattice with non-ionic interactions [13]. Over the past few years, co-crystals have shown significant results in drug development, especially in modifying APIs’ physicochemical properties and pharmacokinetics, such as solubility and dissolution rate, bioavailability, morphology, particle size melting point, physical form, biochemical stability, and permeability [14,15]. In addition, several studies regarding the application of co-crystals in drug delivery studies have been published [16].

Moreover, the formation of co-crystals can increase the analgesic activity of the ibuprofen–nicotinamide (IBU–NIC) co-crystal, studied in vivo by Yuliandra et al. in male Swiss-Webster rats. Based on these tests, the IBU–NIC co-crystal increased the level of pain inhibition by two-fold, i.e., it can reduce pain better than IBU and its physical mixture [17]. In another publication, the ketoprofen–nicotinamide (KET–NIC) co-crystal had the potential to increase the elimination of intracellular pathogens because KET–NIC can increase the production of hydrogen peroxide (H_2_O_2_), which is produced by phagocytes to eliminate intracellular microorganisms, such as Leishmania spp., *Histoplasma capsulatum*, and *Pneumocystis jirovecii*. Furthermore, based on a cytotoxic assay using a colorimetric method, KET–NIC could improve cell survival compared to KET [18]. More recently, crystal engineering has become a promising trend in modulating drug physicochemical properties and, in some cases, could improve the activity [19].

In this review, we report that the manufacture of co-crystals can be categorized into two main techniques, namely solution-based and solid-based methods. The conventional solution-based methods include fast evaporation, slow evaporation, and slurry methods. Moreover, supercritical or gas anti-solvent co-crystallization using CO_2_ were also reported recently. On the other hand, solid-based procedures are considered more efficient because they reduce solvent usage significantly. For example, neat grinding and co-melting using heating can be performed without solvent. Meanwhile, solvent-dropped grinding and microwaving only need a minimal amount of solvent. NSAID co-crystal development, its challenges, and procedures will be discussed in this manuscript, respectively.

## 2. Co-Crystal

**Figure 1:**
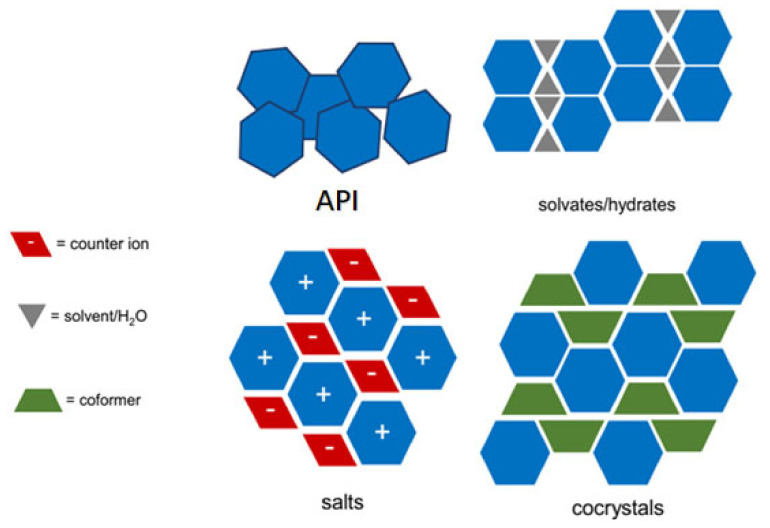
Different solid multicomponent pharmaceutical forms for API. Adapted with permission from ref. [21]. Copyright 2017 Elsevier.

Various experts have discussed the definition, but it is still challenging to distinguish between co-crystals, salts, and solvates/hydrates [20]. Besides, in the single-form, API may form multicomponent crystals, as shown in Figure 1 [21]. 

Historically, co-crystals were first reported by Friedrich Wöhler in 1844 and successfully characterized in 1968 [22]. However, the term co-crystal itself was only introduced in 1963 by Lawton and Lopez. Co-crystals are classified as multicomponent crystals that consist of APIs and co-formers with non-covalent bonding intermolecular interactions such as hydrogen bonds, van der Waals bonds, halogen bonds, and π-π stacking interactions. Repeated intermolecular interactions between functional groups in a crystal involving proton donors and acceptors form supramolecular synthons, which may be composed of the same or different functional groups, named as homo- and hetero-synthons [12].

The number of hydrogen interactions may represent the solubility of the co-crystals. This phenomenon is supported by the hydrotropic effect of co-formers such as proline (PRO) to dissolve a more hydrophobic API with excellent solubility [23]. Additionally, co-crystals show increased dissolution, bioavailability, stability, and mechanical properties. The FDA initially classified co-crystals as intermediate medicinal products in complex molecular APIs and excipients [24] but has now revised its guidelines. A co-crystal is classified as a specific solvate product similar to a polymorph in API. Co-crystals are determined based on the ΔpKa rule, where a pKa value > 1 indicates the occurrence of proton transfer, which produces salt or salt co-crystal. Co-crystals that do not transfer proton are characterized by a pKa value < 1 [13]. Moreover, for co-crystal salts, there is partial proton transfer with a pKa between 1–3 [25]. A salt co-crystal is an ionic co-crystal formed from organic molecules and ionic salts in the form of cation halides [14].

Several publications have reported that salt co-crystals can be generated from the salt form of API and the co-former, but the other components are neutral [21]. Furthermore, the co-crystal can be determined based on the length of the CO bond of the carboxylate group, the dihedral angle, and the CNC angle, where deprotonation will cause the CO bonds in the carboxylate groups to be similar to each other. In contrast, if the CO bonds are significantly different, it indicates protonation [26,27]. The bigger the CNC angle, the higher the protonation degree, causing a higher pK value [28]. For example, the CO distance in paracetamol (PCM)–Oxalic Acid (OXA) is 1.03 and 1.04, while in PCM–Maleic Acid (MLA), it is 1.056 and 1.08. A similar CO distance indicates no proton transfer, so the co-crystal formed is not a salt co-crystal [29].

Commonly, Fourier transform infra-red (FTIR), thermal analysis (differential scanning calorimetry/DSC, differential thermal analysis/DTA, and thermogravimetry/TG), powder X-ray diffractometry/PXRD, and single-crystal X-ray diffractometry/SCXRD are used to confirm the presence of a new solid phase. Based on the IR spectrum, a shift in the C=O peaks of the two co-crystals, indicating no proton transfer to carboxylic acids [30,31] and decreases in the frequency, showed that the functional groups play a role in the formation of strong hydrogen bonds. Several analysis methods using FTIR have been developed, including the multivariate curve resolution alternating least squares (MCR–ALS) method developed for IBU–NIC co-crystal analysis. Analysis by MCR–ALS is a chemometric method for data analysis performed by outlining the IR spectrum pattern in a single component [32].

Thermal analysis with TGA and DSC is an adequate method to detect the new solid phase formation. The sharp thermogram indicates the purity and homogeneity of the co-crystal and good thermal stability [30]. DSC analysis exhibits the unique thermal melting point of the co-crystal, which differs from those of the parent drugs [31,33]. The differences in diffraction peaks and intensity in PXRD inform the new crystal interaction, i.e., interactions between the oxygen atoms (O) and the H atoms of primary amide groups, which SCXRD can structurally determine [31,34]. A change in the solid form indicates crystalline formation, which SEM can characterize [34]. Computational studies have shown co-crystal interactions through the calculation of free energy using Schrodinger software. For example, piroxicam (PXC)–sodium acetate (SAT) co-crystal was reported to have lower free energy (−1671.29 kJ) compared to PXC (−1442.71 kJ) and SAT (−228, 49 kJ). Low free energy indicates that the co-crystal is stable [33].

## 3. Progress on NSAID Co-Crystals

NSAID co-crystals were first reported and patented in 1973 by Gerhard Dorler and Maria Kuhnert-Brandstetter. Over the decades, NSAID have been developed into co-crystals, salt co-crystals, co-crystal hydrates, salt hydrate co-crystals, and drug–drug co-crystals. For example, a co-crystal consisting of two APIs, namely pyrithyldione as sedative and propyphenazone as NSAID, produces three polymorphs; the polymorph form II was the most stable [35]. Since then, NSAID co-crystals have continued to develop in terms of screening, manufacturing techniques, and enhancement of physicochemical properties [23,28,36,37,38]. Furthermore, some invented NSAID co-crystals are summarized in Table 1.

### 3.1. Screening NSAID Co-Crystals

**Figure 2:**
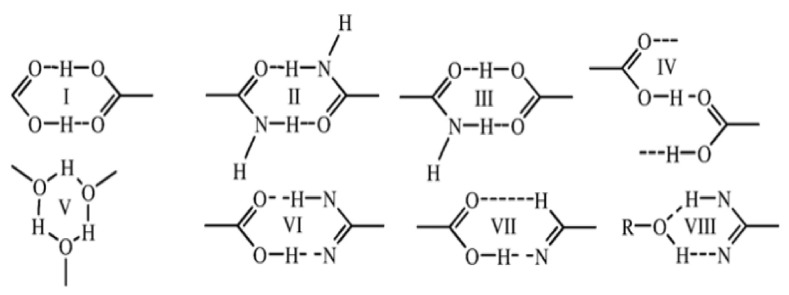
Strong hydrogen bonds in homo-synthons and hetero-synthons. Reprinted with permission from ref. [84]. Copyright 2007 Elsevier.

NSAID co-crystals exhibit various supramolecular synthon motifs (Figure 2) [84]. For example, diflunisal (DIF) co-crystal has three hydrogen bonding patterns based on the Cambridge Structural Database (CSD), namely (1) a ring hetero-synthon with aromatic N, (2) a COOH-COOH homo-synthon, and (3) four ring homo-synthons between OH and C=O from acids. Interestingly, DIF and other salicylic acid (SA) derivatives have an ortho-hydroxy group with steric resonance and hydrogen bonding called the “ortho effect”. Only eight DIF co-crystals have been created with the following features: (a) a COOH homo-synthon between acid co-formers and o-hydroxy carboxylic acids when one of them has an electron-attracting group, (b) COOH homo-synthons between acid co-formers and o-hydroxy carboxylic acids when the o-hydroxy acid has a competing group, such as OH or NH_2_, which can form hydrogen bonds with the co-former, and (c) a combination of motifs (a) and (b) with a COOH interaction distance of more than 3 Å; the type (c) motif is rare. Based on their feature exploration, the ability of donors is in the following order: COOH > NH > OH with the most interactions found in DIF with COOH [38]. Indomethacin (INC) also forms a co-crystal with the saccharine (SAC) co-former by forming N–H·· O hydrogen bonds between acid dimers in INC and imide dimers in SAC [48].

**Figure 3:**
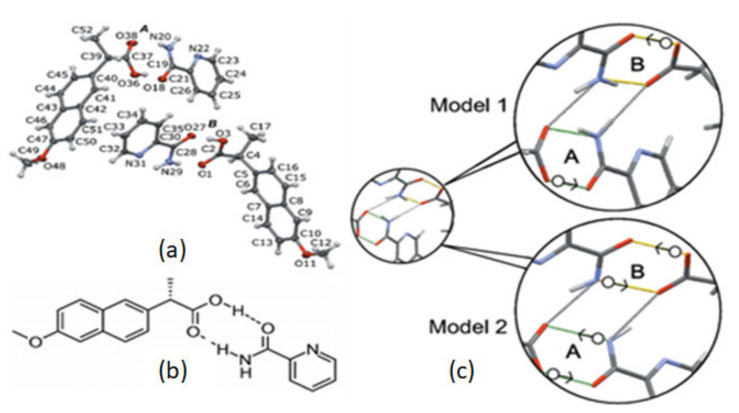
(**a**) Crystal structure of SCXRD; (**b**) synthon formed from NPX–PA; (**c**) H atomic displacement scheme. Reprinted with permission from ref [85]. Adapted from ref. [85]. Copyright 2017 NCBI.

Naproxen (NPX)–picolinamide (PA) co-crystal was formed from carboxylic acid-carbamide dimers between NPX and PA, as shown in Figure 3. PA offers an ortho effect with a pKa value of 1.17. For that PA, carboxamide interacts with NPX via acid-amide synthesis; however, SCXRD results do not match the asymmetric unit. The position of the proton determined by H nuclear magnetic resonance (NMR) shows that the H atom involved in hydrogen bonding at the synthon gives a different peak. The H atom involved has a delay of 20s compared to the other H atom, which was determined by comparing the NMR data with the predicted shift values. The RMSD obtained was 0.09 Å by optimizing the atomic position at 0.2 Å SCXRD so that the hydrogen bonds are more symmetrical and the difference between the two hydrogen bonds O–H · O was reduced. 

In Figure 3c, two bonding models are proposed as alternatives to improve the SCXRD results. In model 1, only the H atoms in the OH bond are removed, whereas, in model 2, the amide protons in PA also move along the N–H hydrogen bond. H atom position was validated by density functional theory (DFT) geometry optimization based on a significant decline in potential energy, which indicates that the naproxen–picolinamide (NPX–PA) is a co-crystal and not a salt [85].

Lornoxicam (LNX) is included in the oxicam class of NSAID drugs. LNX has more selective anti-inflammatory activity without affecting the digestive tract. LNX can be used for osteoarthritis therapy [86] and postoperative pain relief of the head and neck, and also reduced postoperative pain in the third molars in the first phase of perioperative pain management [87]. LNX has better tolerance and safety than tramadol and is comparable to tramadol in postoperative analgesic therapy [88]. Oral administration of LNX takes 2.5 h to produce a maximum concentration (Cmax), limiting LNX therapy for providing the optimal effect as an analgesic. The co-crystallization technique has succeeded in changing the physicochemical properties of LNX using the liquid-assisted grinding (LAG) method. From this method, co-crystals of LNX salt were produced with a benzoic acid co-former and 2,4 dihydroxy benzoic acid. 

In contrast, co-crystals were formed from catechol co-formers, resorcinol, hydroquinone, and SAC at a stoichiometric ratio of 1:1 [77]. The crystal system is orthorhombic with space group P_212121_ with one LNX molecule in asymmetrical units based on the synthon co-crystal approach. The decrease in the melting point of the parent drug indicates the formation of a multi-component system. Strong hydrogen bonds link the LNX N^+^–H···O=C and NH···OC causes a decrease in the conformational change in the amide bond to the ring. As a result, a zigzag band formed along the C axis in the molecule through the interaction of N^+^–H···O=S, C–H···O and C–H···Cl, then the CH···S interaction forms a 2D sheet and creates a 3D layered structure through CH···O hydrogen bonding interactions. Based on in vitro dissolution testing at the optimum temperature of LNX, the salt co-crystal has excellent solubility compared to the co-crystal and LNX, i.e., up to 1.52-fold higher than that of LNX [78].

LNX co-crystals were also formed by the neat grinding method on the generally recognized as safe (GRAS) co-former and drugs such as malonic acid (MAL), succinic acid, tartaric acid, anthranilic acid (phenamate), cinnamic acid, p-aminobenzoic acid, ferulic acid, urea (URE), sodium saccharin (SS), citric acid, and OXA. LNX–SS increases the solubility of the parent drug significantly. It is related to the low lattice energy value, thereby reducing the interference from the solvent. Co-crystallization decreases the partition coefficient and shows the transformation from a hydrophobic to a hydrophilic compound. The LNX–SS co-crystal has good stability at extreme temperatures based on stability testing for 30 days under 40 °C and 75% humidity [76].

**Figure 4:**
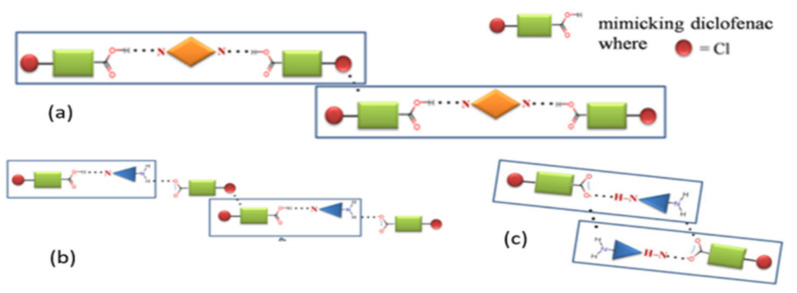
(**a**) Packing of trimer synthon aggregate III in co-crystals 1, 2, and 3; (**b**) aggregation connecting synthon IV; (**c**) and packing of tetramer synthon aggregate IV. Adapted with permission from ref. [25]. Copyright 2020 Elsevier.

Co-crystal diclofenac (DFA) with pyridine-based co-formers and acid pyridine synthon has been reported in nine new solid forms using the evaporation co-crystallization technique. DFA formed co-crystals with the 1,2-bis (4-pyridyl) ethane (BPE), 1,3-di (4-pyridyl) propane, and 4,4-bipyridine at a stoichiometric ratio of 2:1. The three co-formers have pyridine groups on both sides. The packing of DFA co-crystal is highlighted in Figure 4a,b. Simultaneously, DFA formed salt co-crystal with two amino groups as co-formers, namely 2-aminopyridine (2-apy) and 3-aminopyridine. In the salt co-crystal DFA-2-apy, there is a weak bonding of C–H. There is a tetramer aggregate packaging in the salt co-crystal that connects the synthon, as shown in Figure 4c [25]. 

The formation of NSAID co-crystals with an amino acid as the co-former has been widely published, called zwitterionic co-crystals. For example, indomethacin–proline (INC–PRO) 2H_2_O, a co-crystal hydrate, exhibited the supramolecular synthons NH^3+^···O^−^ and OH···O. The INC–PRO co-crystal consists of an internal hydrophilic group and a hydrophobic group on the surface, increasing the solubility and permeability INC [23]. Besides INC, a diclofenac–proline (DFA–PRO) was obtained as a stabilized co-crystal and linked with intramolecular hydrogen bonds N–H···O and C–H···C. The difference in the C–O bond length of the DFA and PRO carboxylate groups > 0.07 indicates that DFA–PRO is a zwitterion co-crystal [48,49].

Furthermore, Nugrahani et al. developed the DFA–PRO co-crystal by forming a salt hydrate co-crystal to increase the multi-component solubility and stability over DFA–PRO co-crystal and the alkaline salt diclofenac. Besides sodium diclofenac proline tetrahydrate (SDPT), a co-crystal of sodium diclofenac–proline monohydrate (SDPM) was confirmed by a structural determination with SCXRD at −180 °C. The new phase consisted of diclofenac sodium–proline-water (1:1:1:1). A stability study of the two co-crystals was carried out under extreme drying and humidity and evaluated by differential thermal analysis (DTA)/TG/DSC). Based on the diffractogram results, SDPT was stable at high humidity, i.e., 94 ± 2%RH/25 ± 2 °C, for up to 15 days did not separate into the starting materials [48].

Meanwhile, SDPM quickly changed to SDPT under these environmental conditions. Moreover, SDPM could be restored to SDPT under dry conditions, so SDPT is a more stable co-crystal than SDPM. These results show that water plays a critical role in forming co-crystals in SDPT to mediate the interactions between the components in the co-crystals of the tetrahydrate salt formed. The high solubility of SDPM occurs because there is a region consisting of Na^+^ and water molecules with high affinity, such that it quickly dissolves and breaks down. The dissolution test was carried out at pH 1.2 and 6.8, based on previous studies; the sodium diclofenac (SD) profile increased at moderate to alkaline pH. Based on this test, the SDPM and SDPT dissolution results were faster than SD, with a superior increase in the dissolution of SDPM. This result showed that the monohydrate crystal lattice was smaller and had a looser and broader space than the tetrahydrate form, so it dissolved faster than SDPT at pH 6.8 [27]. Furthermore, the DFA–PRO co-crystal was developed from diclofenac potassium, resulting in a salt co-crystal hydrate consisting of potassium, DFA, l-proline, and water (1:1:1:4) [51].

The prediction of bond formation between co-crystal NSAID and co-formers using a computational method to calculate DFT with B3LYP/aug-cc-pVDZ 1 depends on the constituent molecules to form a supramolecular synthon [44]. Co-crystal screening with various co-formers and methods has been established, one of which is by calculating the van Krevelen and Hoftyzer solubility parameters, involving the solubility of two compounds to find the ∆δ^−^ factor with the equation:

∆δ^−^ = [(δ_d2_ − δ_d1_)^2^ + (δ_p2_ − δ_p_)^2^ + (δ_h2_ δ_h1_)^2^]^0.5^ (1)

The partial solubility parameters are dispersion (δ_d_), polarity (δ_p_), and hydrogen bonding (δ_h_). Good miscibility can be achieved if ∆δ ≤ 5 MPa^0.5^. The difference in total solubility between the drug and the ∆δt carrier is a means of predicting solubility:

∆δ_t_ = |δ_t2_ − δ_t1_| (2)

As noted, t1 and t2 are carriers and drugs; materials are miscible with ∆δ ≤ 7 MPa^0.5^, while systems with ∆δ ≥ 7 MPa^0.5^ are immiscible. This solubility parameters method was used for developing aceclofenac (ACF) co-crystals with several co-formers, namely gallic acid (GLA), CA, MLA, NIC, d-tartaric acid (TCA), URE, and vanillic acid, which showed that all co-formers could form co-crystals, except TCA [31]. ACF 2-[2-[2-[(2,6-dichloro-phenyl)amino]phenyl]-acetyl] oxy-acetic acid (C_16_H_13_C_l2_NO_4_) is an analog of DFA in the form of glycolic acid ester. It is used as a first-line drug for the treatment of rheumatoid arthritis and osteoarthritis [89]. The gastrointestinal side effects of ACF are relatively lower than other non-selective NSAID and are comparable to CEL [31]. ACF does not interact directly with COX enzymes, but ACF in the form of a prodrug will produce DFA active metabolites, which will inhibit COX enzyme activity by forming hydrogen bonds on the TYR355 and SER530 residues of COX enzymes [90]. The characterization of the ACF–NIC and ACF–GLA co-crystals obtained by the solvent evaporation technique at 1:1 stoichiometry was based on the SEM analysis. The ACF–GLA and ACF–NIC co-crystals had different crystal shapes than pure ACF, large and regular, while the co-crystals had a more irregular shape [31]. Based on the FTIR structure, five hydrogen bonding motifs arranged the ACF–NIC co-crystal, which is (1) the formation between the amide group from NIC and the acid group from ACF, (2) hetero-synthon chloride-amide formation, (3) ACF linked to NIC by an acid–pyridine hetero-synthon (synthon I) or acid–amide hetero-synthon (synthon V), (4) amide–amide dimers by NIC and ACF groups bound to an acid-amide hetero-synthon, and (5) ACF formed by synthon I and an amide–chloride hetero-synthon [53].

### 3.2. Development of NSAID Co-Crystal Production

In developing co-crystals, the method is crucial to produce the expected physicochemical properties, as demonstrated by the indomethacin–proline (INC–PRO) system, produced by LAG, and solvent evaporation. The LAG method yielded polar molecules, thereby improving the solubility and intrinsic dissolution rate (IDR) under each tested pH condition; high solubility indicates increased bioavailability and may improve the pharmacokinetic profile of INC [23]. Jafari et al. categorized the co-crystal production route in two ways, solution and solid-state based. Evaporative co-crystallization, cooling crystallization, reaction co-crystallization, isothermal slurry conversion, and supercritical antisolvent (SAS) are solution-based production methods. Conversely, the solid-state process is a technique to combine the solid materials directly, and pressure is applied manually (mortar and pestle) or mechanically (automatic ball mill).

**Figure 5:**
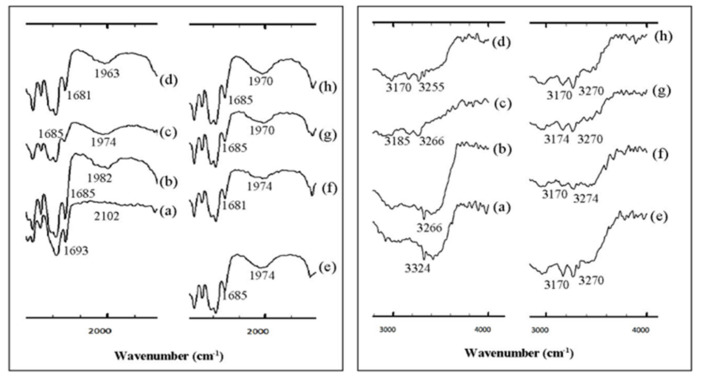
DFA FTIR spectrum (left) and DFA–PRO co-crystal (right) with the LAG method in (**a**) 0 min; (**b**) 2 min; (**c**) 4 min; (**d**) 10 min; (**e**) 15 min; (**f**) 30 min; (**g**) 60 min; (**h**) and 90 min. Note: DFA = Diclofenac; PRO = Proline. Reprinted with permission from ref. [49]. Copyright 2018 Elsevier.

**Figure 6:**
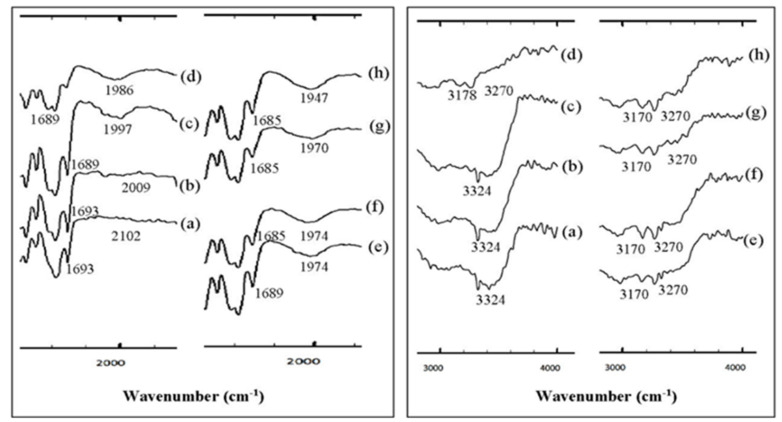
DFA FTIR spectrum (left) and DFA–PRO co-crystal (right) with the NG method in (**a**) 0 min; (**b**) 2 min; (**c**) 4 min; (**d**) 10 min; (**e**) 15 min; (**f**) 30 min; (**g**) 60 min; (**h**) and 90 min. Note: DFA = Diclofenac; PRO = Proline. Reprinted with permission from ref. [49]. Copyright 2018 Elsevier.

The most common solid-state methods are neat (dry) grinding (NG), LAG, and hot-melt extrusion. The direct solid-state process significantly reduced solvent usage, so it is preferable in the context of the evergreen method [91]. However, the different techniques may not produce the same form and physicochemical properties, evidenced by several publications. For example, Evora et al. reported a co-crystal with three methods: annealing a mortar ground mixture at 80 °C, annealing at room temperature after neat ball mill grinding, and ethanol-assisted (10 μL) ball milling. The neat mill grinding methods produced only a few crystals. Meanwhile, the annealing mixture at 80 °C formed the new solid phases with a melting point at around 113–146 °C. In this study, eight co-crystals were created with the ball milling method. In contrast, the diflunisal (DIF) co-crystal with pyrazine, which has a crystalline form like a diamond, was formed by a crystallization method using a solution. DIF can be crystallized with a greener solvent with OXA as the co-former [87]. The solution method was then compared to LAG with ethanol and NG. Observation with FTIR at a specific time showed significant changes in the spectra (Figure 5 and Figure 6) [49].

With a longer grinding time, the specific peak transformation of the co-crystal was more apparent as the peak of the parent compound decreased and disappeared. The regions at wavelengths 1500–2500 cm^−1^ and 2500–4000 cm^−1^ show the specific evolutionary areas on the formation of the co-crystal, which indicates a gradual shift of the carbonyl groups to a lower wavenumber, i.e., from 1693 cm^−1^ to 1685 cm^−1^ after 30 m of the grinding process. This phenomenon is related to the decreased vibrational energy of the carbonyl, which is caused by the formation of heterocyclic N hydrogen bonds with the PRO group. Besides the carbonyl, the OH peak also experienced a shift from 3324 cm^−1^ to 3270 cm^−1^ accompanied by a new band at 3170 cm^−1^, estimated from the OH carbon oxide group DFA which forms hydrogen bonds with the PRO carbonyl group. Therefore, this shift was accompanied by a change in the PRO carbonyl group from 1652 and 1617 cm^−1^ to 1623 and 1616 cm^−1^. The new peak appeared at 1968 cm^−1^ due to the presence of hydrogen bonds O···H–N, which occurred after 2 min of grinding and formed a sharp spectrum after 60 min of grinding. The specific peaks of the co-crystal appeared regularly at 3270 and 3170 cm^−1^. The DFA–PRO co-crystal dynamics using the NG method showed the same FTIR pattern as the LAG method; however, the initial co-crystal formation took longer in co-crystallization using the NG method, i.e., within 10 m, while with the LAG method, the initial co-crystal was formed within 2 min. This result is consistent with previous research showing that the addition of solvents will increase molecular diffusion, thereby increasing the interaction between the API and co-formers [49].

Sevukarajan et al. successfully synthesized an ACF–NIC (1:1) co-crystal with the NG method which showed a better solubility because it produced smaller crystals than the solvent evaporation (SE) method [43]. Besides offering a different solubility, different co-crystallization methods also yield various crystalline forms. Berry et al. obtained two co-crystal phases of ibuprofen–nicotinamide (IBU–NIC) by melting and slow evaporation method, namely the rectus-sinister ibuprofen–nicotinamide (RS-IBU–NIC) and sinister ibuprofen-nicotinamide (S-IBU–NIC) [92]. Guerain et al. also studied the formation of IBU-NIC co-crystal with different co-crystallization methods: (1) milling, (2a) crystallization by melting the mixture at 100 °C and then cooling to room temperature, (2b) crystallization by melting the mixture at the glass transition temperature, and (3) the slow evaporation of the solvent. After being characterized by PXRD, each co-crystallization method produced different S-IBU–NIC co-crystals [93]. Methods (1), (2b) and (3) resulted in a similar S-IBU–NIC found by Berry et al. In contrast, co-crystallization with a process (2a) resulted in a new S-IBU-NIC co-crystal. The stability of the new S–IBU–NIC co-crystal showed transformation to the previous S-IBU–NIC co-crystal at temperature 65 °C. In this study, the crystallization method by heating close to the melting temperature will produce polymorphisms [93].

Mefenamic acid (MFA) can arrange co-crystals with several co-formers, such as NIC, URE, pyridoxine, etc. Of all the co-formers reported, NIC was selected as the co-former used in a study on co-crystal formation using the melt crystallization technique because contaminants can form when MFA is co-crystallized by a grinding process. This technique was carried out by melting MFA with NIC (1:2) in a porcelain cup over a paraffin oil bath with the temperature maintained at 200 °C, then incubating in a container containing water over a water bath with the temperature maintained at 90 °C, then drying at room temperature overnight. Based on the characterization results with PXRD, FTIR, DSC–thermogravimetric analysis (TGA), and thin-layer chromatography (TLC), the heating process did not cause co-crystal decomposition. Moreover, from the solubility test results, the solubility of MFA–NIC was higher than that of the parent compound [64].

**Figure 7:**
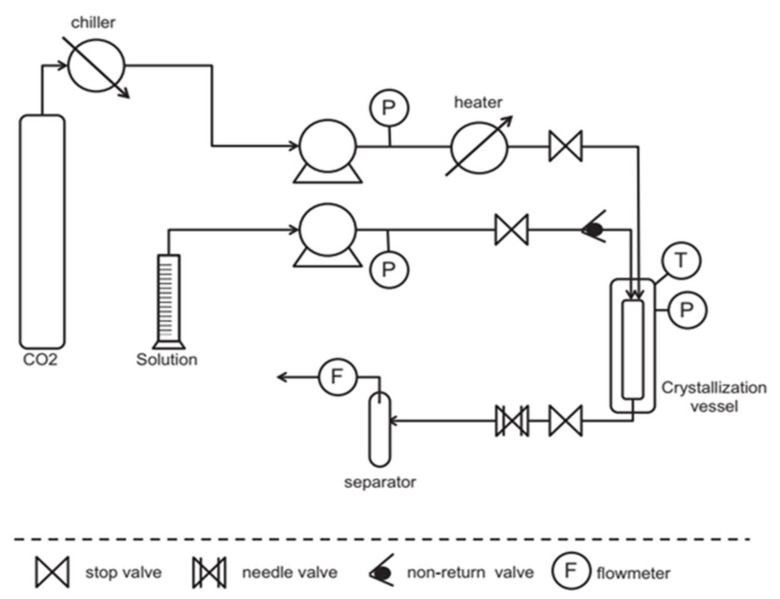
Schematic diagram of SAS technical setup. Reprinted with permission from ref. [57]. Copyright 2016 Elsevier.

The supercritical anti-solvent (SAS) method has been used in the formation of NSAID co-crystals. The principle of this method is to dissolve the API and co-former until saturated with the appropriate solvent. The addition of CO_2_ reduces the solubility of the API and co-former to induce precipitation to produce NSAID co-crystals [91]. SAS methods can also compose naproxen (NPX)–NIC co-crystals at an equimolar ratio of 2:1 with a solvent mixture (acetone and CO_2_) subjected to high pressure (10 MPa) at 298.15 310.65 K [94]. Neurohr et al. also researched NPX co-crystallization techniques besides conventional co-crystallization techniques, namely the SAS technique, as shown in Figure 7. PXRD and FTIR analysis concluded that the SAS technique produced NPX–NIC co-crystals with the same characteristics as conventional co-crystallization techniques [57].

Wichianphong et al. formed MFA–NIC co-crystals using the gas anti-solvent (GAS) method under conditions optimized with the Behnken experimental design. Parameters that can affect the process were investigated, such as temperature (T), the molar ratio of the co-former to the drug (C), and the percentage of drug saturation (S) in the solution against t_63.2_ (the time required to dissolve 63.2% of the drug). The resulting co-crystals were then compared also with the product from the conventional methods and physical mixtures. Based on the difference in melting point, PXRD pattern, and FTIR spectrum, the formation of a new phase was confirmed, namely the MFA–NIC co-crystal. The fastest dissolution time (5.07 min) at 450 °C, a co-former-to-drug ratio of 5:1, and 70% drug saturation. The t_63.2_ obtained from the experimental results and the calculation of the equation was suitable and met the requirements of (R^2^ ≥ 80%) with R^2^ amounting to 96.25% and 89.51%; hence this model provides a good correlation. ANOVA showed that S and C significantly affected the t_63.2_ (*p* < 0.05 and value of F = 14.27) where, at low T, t_63.2_ could reach the minimum value if S and C were high, which can increase the S value. A high S value can prevent the formation of MFA–NIC co-crystals and result in a faster dissolution time [65].

**Figure 8:**
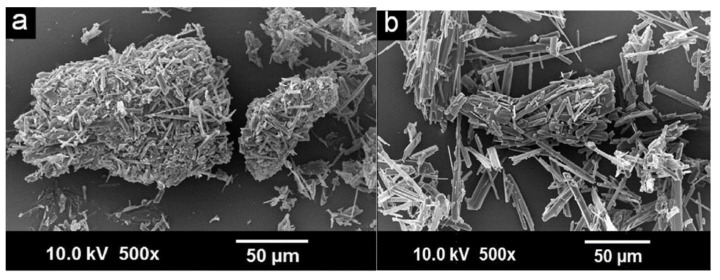
Microscopic observations of MFA–NIC co-crystallization results with the (**a**) conventional method and (**b**) the GAS method. Reprinted with permission from ref. [65]. Copyright 2018 Elsevier.

**Figure 9:**
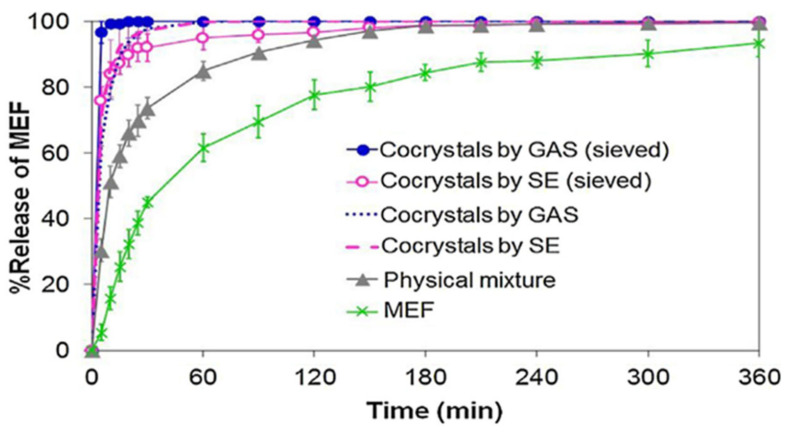
The dissolution profile of MFA–NA which was co-crystallized by the conventional and GAS methods. Reprinted with permission from ref. [65]. Copyright 2018 Elsevier.

In addition to DSC, PXRD, and FTIR, the co-crystal MFA–NIC co-crystallization results by the GAS method were also characterized by SEM. Observations showed that the form of MFA–NIC was different from the parent compound and the co-crystal produced by the conventional method (Figure 8). From the dissolution test, the co-crystals from both methods provided an increase in the dissolution rate (Figure 9) [65]. The advantage of this method is a uniformly shaped co-crystal with high purity due to the filtration process with a particular size and a washing process with CO_2_ to remove the remaining solvent in the MEF–NIC co-crystal [65]. In addition, this SAS technique depends on the composition of the fluid used and can cause heterogeneity [57].

As the development of technology in pharmaceutical engineering increases, the co-crystallization technique continues to be developed to improve solubility and permeability, one of the premises of applying nanotechnology in co-crystallization. Nanotechnology is the most established technology for increasing the therapeutic index and overcoming the challenges of formulating compounds with poor solubility. From several studies, the nano co-crystal approach has been shown to increase the solubility and dissolution of drugs with poor solubility. This result is related to the size of the co-crystal being reduced to nano-size (less than 100 nm), increasing the surface area and the dissolution rate. Increased dissolution will be very useful to enhance bioavailability. Moreover, nano co-crystal engineering can reduce toxic solvents and surfactants and develop a formulation for various routes of administration where size is a critical factor (injection, ophthalmics, and topicals) [95].

The nanocrystal mineral has limitations, including that it can only be used for class II BCS drugs, requiring high-cost instruments, with formation and stability depending on the drug molecule. Only certain compound groups meet the requirements. A large nano-co-crystal surface with high free energy or differences in surface charge can cause aggregation. However, an increase in solubility that exceeds the saturation point will cause recrystallization into larger particles, called Ostwald refining. Selecting a stabilizer in the formulation of a nano-co-crystal preparation to protect the particle surface in order to reduce the free energy of the system and the interface voltage of the nano-co-crystal overcame this occurrence [96].

The nano–diclofenac–proline co-crystal (NDPC) is formed by combining two techniques, i.e., top-down with the NG method and bottom-up with microwave-assisted rapid evaporation. The production of NDPC with NG produced pure nano-co-crystals with a particle size of around 857.9 nm with a polydispersity index (PI) of 0.353 after 6 h. However, there are limitations to this method related to co-crystal instability. The best sized NDPC at 598.2 nm and PI 0.278 was successfully obtained by the fast evaporation method with ethanol as the solvent and 776 W microwave energy in 8–12 min. Microwave energy provides molecular rotation to form intermolecular bonding interactions in the co-crystal. In stabilizing NDPC, sodium lauryl sulfate (SLS) was added as a stabilizer, giving a zeta potential value of −660 mV, indicating the particles are stable without forming agglomeration. In addition, SLS provides a negative charge on the zeta potential to disperse the nano co-crystal solution [52].

A scale-up process was carried out to produce pure crystals on the scale of 10 g using the refrigerant crystallization method without seeding (adding crystal seeds), equipped with a temperature sensor. This method used a heating and reflux technique to dissolve the co-crystal in ethyl acetate, slowly lowering the temperature with an optimized stirring cycle. Based on PXRD and DSC analysis, the scaled-up indomethacin–saccharine (INC–SAC) co-crystal product has the same purity with the yields of a small scale as the solvent evaporation method using ethyl acetate [47]. Apart from these methods, a combination method (SAS and cooling co-crystallization) could scale up the INC–SAC co-crystal. Cooling time is a factor for optimal production conditions, as cooling will accelerate the precipitation of the co-crystal. The post-nucleation cooling time resulted in a greater amount of INC–SAC co-crystal with smaller particle sizes [97].

### 3.3. Enhancement of the Physicochemical Properties of Co-crystals

**Figure 10:**
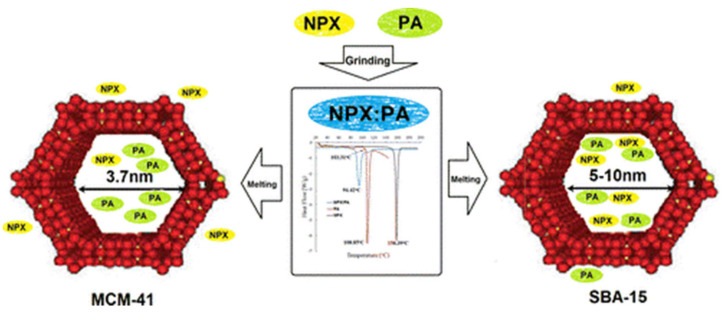
The thermal solvent-free method to include NPX–PA co-crystals into MSP. Reprinted with permission from ref. [36]. Copyright 2016 American Chemical Society.

Based on studies conducted by Skorupska et al. to increase solubility and prevent degradation, modified drug delivery was studied by inserting the naproxen–picolinamide (NPX–PA) co-crystal into mesoporous silica particles (MSP) using the thermal solvent-free method, by heating a mixture of co-crystal and MSP at 100°C for 2h. The complex NPX–PA co-crystal in MSP as shown in Figure 10 was prepared to protect the API from environmental effects, carry the drug across the cell membrane, accelerate the drug’s action, increase treatment efficiency, and deliver the drug to specific target organs. Two MSPs with different pore sizes were used; NPX–PA was successfully inserted into SBA-15 (100 Å), while MCM-41 (37 Å) acted as a separating medium. NPX stuck to the outer wall of MSP since the pore size of MCM-41 is smaller than NPX, so SBA-15 is more suitable for the NPX–PA co-crystal [36].

Sohrab et al. conducted a study to observe the effect of a water-soluble polymer on aceclofenac–nicotinamide (ACF–NIC) co-crystal 1:1 obtained from the solvent evaporation and NG methods. PVP K30, hydroxypropyl methylcellulose (HPMC), sodium starch glycolate, and carboxymethylcellulose sodium (CMC-S) were mixed with the co-crystals, and the mixture was then tableted by the wet granulation method. The addition of water-soluble polymers lowered the melting point of the co-crystals, decreased the lattice energy, and thus increased the dissolution rate of ACF. Based on dissolution testing with the USP type I method in phosphate buffer pH 7.5 medium, the ACF–NIC co-crystal without the addition of 3% water-soluble polymer showed 78.9% and 78.5% drug release, while the co-crystal formulations MUNG01 (3% PVPK-30), MUNG02 (3% HPMCE5), MUSE03 (3% SSG) and MUSE04 (3% CMC-S) showed maximum release of 99.1%, 97.51%, 99%, and 98.25% [98].

Next, ACF was also developed into a topical formulation, which was expected to have a high penetration rate through each layer of the skin to effectively and safely treat pain locally. Sharma et al. developed a nanoliposome formulation of ACF with amino acid lysine (LYS) to increase the co-crystal penetration to the skin. ACF–LYS was encapsulated using liposomes formed from lipids and cholesterol in a 70:30 ratio, then dissolved in chloroform and evaporated. ACF–LYS was suspended in the liposome and then inserted into a vehicle from carbopol 940 hydrogel to increase viscosity and form a translucent gel. The co-crystal-loaded liposome gel (COC–LG) formulation, with an average size of 120.4 ± 1.03 nm, showed greater drug penetration into the deeper skin layers. The co-crystal had a spherical shape based on imaging studies with confocal laser scanning microscopy and transmission electron microscopy. The COC–LG formulation had a lower viscosity than the gel on the market, which indicated good contact time and dispersion, resulting in maximum therapeutic effect. Based on ex vivo testing using the back skin of Wistar rats, the formula penetrated better and remained in the skin 2.31 times longer than the marketed gel. The penetration increase was associated with the interaction of the biocompatible component (phospholipon) with the skin. The COC–LG formulation increased the drug concentration in a short time, did not interfere with skin integrity, did not cause inflammation of the dermal tissue (as did other gels), and was more effective as an analgesic and anti-inflammatory for the treatment of arthritis and osteoarthritis [55].

Co-crystallization can also consist of APIs, metals and organic materials to accelerate NSAID action and prolong the half-life, thereby increasing the drug’s duration of action. Hartlieb et al. formed an IBU–metal–organic co-crystal called a metal–organic framework (MOF). MOF is a γ-cyclodextrin (γ-CD) metal used in the form of cation metals such as potassium (K^+^) or sodium (Na^+^). CD–MOF was used to create an organic porous framework filled with IBU. The co-crystals produced by the diffusion of ethanol vapor in a CD solution and ibuprofen potassium salt formed a cyclodextrin metal–organic framework (CD–MOF-1) co-crystal with an IBU loading rate of 23% in the CD–MOF-1 co-crystal. One problem with IBU is the active S-enantiomer and less active R-enantiomer. However, IBU was absorbed by CD–MOF-1 as the less active enantiomer, but MOF succeeded in separating the enantiomer from IBU. Co-crystal CD–MOF-1 had better stability in the atmosphere than IBU salt and was not hygroscopic, as seen from the PXRD pattern, where it did not show co-crystal degradation in ambient humidity. Based on bioavailability testing with female rats, the pharmacokinetic data showed that the CD–MOF-1 co-crystal had a Cmax, area under the curve (AUC), and a half-life that was two times higher than that of the IBU salt. This result indicated that IBU absorption was fast with an onset of 10–20 min [99].

### 3.4. Drug–Drug Co-Crystals of NSAID

Co-crystallization of two APIs was also arranged to improve the physicochemical properties of the drug combination. This strategy benefits NSAID drugs, where their therapeutic use is often connected to treat mild to moderate pain or for continued treatment of gout if single therapy cannot be used. Pathak et al. performed co-crystallization techniques on PCM, INC, and MFA drugs with various methods such as solvent evaporation, grinding, the addition of antisolvents, and ultrasound-assisted techniques. Co-crystals were formed from solvent evaporation, which worked best compared to other techniques. The PCM–INC and PCM–MFA co-crystals created hetero-synthon supermolecular synthons with strong hydrogen bonds between COOH–N and COOH–O [83].

The formation of NSAID drug–drug co-crystals can improve drug stability, i.e., the piroxicam (PXC) co-crystal with an aniline–nicotinic acid derivative NSAID, namely clonixin (CNX). Based on stability testing, there was no change in color and transformation into a hydrate at a storage temperature of 25 °C and humidity up to 95% for 4 weeks. Co-crystallization of the PXC–CNX aimed to inhibit the phase transition from the anhydrous to hydrated form, changing the physicochemical and pharmacokinetic properties. CNX was chosen because it has a synergistic therapeutic effect with PXC and CNX structure and has many carboxyl groups to be a co-former. Moreover, it can form both neutral and zwitterionic molecules. In the formation of the PXC–CNX co-crystal, three parameters need to be considered, i.e., the hydrogen bond donor and acceptor’s ability and the polarity of the solvent used. The moderate polar solvents can be used to produce the PXC–CNX co-crystal. Moreover, thermal analysis with DSC showed that the solvent molecule played an essential role in stabilizing the crystal structure. The calculation of the interaction energy with DFT showed that homo-molecular interaction has superior energy than hetero-molecules. Homo-molecular bonds act as a driving force for the decomposition of PXC–CNX compared to a solvent-free co-crystal. Based on screening with the slurry method, there was only a difference in the ethyl acetate (EA) solvent. The PXC–CNX-EA had a different C–H···C distance EA molecule, longer than other solvent molecules. In addition, EA is a non-toxic compound, so that it is safe for food and drug formulations. The crystal structure of PXC–CNX was obtained after the solvent was evaporated and the two molecules were linked via O–H···O hydrogen bonds between the COOH group on CNX and the OH group on the deprotonated PXC. The PXC zwitterion formed hydrogen bonds with the CNX zwitterion via N–H···O, comprising a protonated pyridine donor and a carbonyl amide acceptor [71].

Combined NSAID have better anti-inflammatory activity than single drugs, such as DFA-ethyl diclofenac (ED) co-crystalline, which is more effective than diclofenac acid because only a small amount of ED is bound to plasma. However, the esterification process between the ester and DFA can produce unwanted products. Therefore, a co-crystal between ED and DFA was made, and crystallographic studies were carried out. From the results of thermal analysis with DSC, the melting point of the co-crystal was greater than that of ED (67.7 °C) and lower than that of DFA (173.1 °C), i.e., around 103–104.3 °C. The results showed that the FTIR spectrum did not change. Thin layer chromatography analysis showed the same pattern for the two accelerated stability tests, heated with microwave energy and stored in high humidity for seven days. Thus, the ED and DFA co-crystal were chemically stable. Moreover, anti-inflammatory activity was studied using five groups of mice, which showed that the co-crystals between ED and DFA increased anti-inflammatory activity more than the single components [50].

SCXRD, PXRD, and FTIR characterized crystal meloxicam (MLX) and aspirin (ASP), showing different spectra from the starting material. Based on the rules of pKa, the meloxicam–aspirin (MLX–ASP) co-crystal has not been defined, with a pKa value of 0.68, and a neutral phase with a CNC angle of 110.2°. In a comparable solubility study in the testing medium of phosphate buffer solution pH 7.4 at 37 °C, the solubility of MLX in MLX–ASP co-crystals increased to 0.22 mg/mL from 0.001 mg/mL. Pharmacokinetic studies using male Sprague-Dawley rats at an oral dose of 1 mg/kg showed an increase in absorption of the MLX–ASP co-crystal with a bioavailability of 69%. At the same time, that of MLX was only 16%. The MLX–ASP co-crystal could also achieve tmax four times faster than the single MLX, so it reached the concentration of 0.51 g/mL MLX in 10 min. This rapid onset is an advantage of the MLX–ASP co-crystal, which is indicated as a mild to moderate acute pain reliever. In this study, ASP in the MLX–ASP co-crystal was only 7.7 mg and MLX 10 mg/kg, so it did not cause significant side effects [74]. 

The multi-APIs co-crystal was also constructed between celecoxib (CEL) and tramadol HCl (TML···HCl), named CTC. Initially, the co-crystal was formed by grinding method using isopropyl alcohol. This new phase is an ionic co-crystal, where the chloride ion interacts with an adjacent drug molecule by involving –N^+^ H···Cl^−^ and –OH···Cl hydrogen bonds. The maximum concentration of CEL in CTC was higher than the maximum concentration of a single CEL, thus extending drug action by slowing drug release. Additionally, this combination increased efficacy, preventing pain by four mechanisms of action and lower therapeutic doses, as the CTC dose was 100 mg (44 mg TML · HCl and 56 mg CEL). In contrast, the dose of CEL and TML · HCl is around 200–400 mg/day [79]. Because of its very high efficacy, the co-crystal (TML · HCl–CEL) was tested in a phase I clinical trial in 2017 by Esteve (E-58425) and Mundiphara Research (MR308). In stage I, a single pharmacokinetic (PK) dose of CTC and its reference products were compared singly and in combination. Samples were randomly assigned with an initial dose of 200 mg of CTC, equivalent to 88 mg of tramadol and 112 mg of CEL. The results of the AUC value were comparable to the Cmax, but lower than that of tramadol. However, the t-max was longer than that of tramadol. This result is consistent with previous research where C-max reduction was proportional to the slow dissolution rate [100].

Next, they continued the phase II clinical trial in patients. There were six doses of CTC tested (CTC 50, 100, 150, 200 mg; tramadol 100 mg; and placebo). The initial efficacy of this test was based on differences in pain intensity across the population. Based on the results of phase II, the potential for CTC was more significant than the risk. Furthermore, CTCs at 100, 150, and 200 mg doses were more effective than tramadol 100 mg and placebo for treating moderate to severe acute pain [80]. CTC development is currently awaiting the results of a phase III trial [101,102,103], and this is the first co-crystal NSAID to enter clinical trials, while the MLX–ASP co-crystal is still in the in vivo testing phase [74].

For gout therapy, NSAID are the first-line drugs to reduce pain, which need to minimize uric acid levels in the body by increasing excretion or inhibiting the formation of uric acid using uricosurics and xanthine oxidase (XO) inhibitors [89,90]. To achieve optimal therapy, Modani et al. utilized co-crystallization in combining PXC with febuxostat (FBX). FBX is a novel non-purine xanthine oxidase (XO) used to treat hyperuricemia in gouty patients. Based on several studies, FBX is effective at inhibiting lung inflammation in animals caused by oxidative stress [104,105,106], has been shown to accelerate restoration of the pulmonary endothelial barrier [107], and is potent in treating mild to moderate COVID-19 infection by overcoming the early-phase pneumonia caused by the coronavirus [108]. A single crystal of the piroxicam–febuxostat (PXC–FBX) co-crystal with a stoichiometric ratio of 1:1 resulted from the crystallization technique using acetonitrile solvent. The formation of co-crystals was confirmed by a decrease in the melting point, no stretching of the OH from carboxylic acids indicating no proton transfer, the specific shape, and differences in the diffractogram. A supramolecule synthon connects the carboxylate and azole groups with hydrogen bonds NH···O and 2D packaging, stabilized by the interaction of NH···O, CH···N, OH···O, and CH···O hydrogen bonds. It becomes interesting to learn when both APIs come from the same BCS class. Solubility testing at pH 1.2–7.4 resulted in 2.5 times higher solubility for FBX at pH 1.2 and continued to increase as pH increased; however, at pH 4.5, the solubility decreased. At pH 6.8 and 7.4, the solubility of PXC significantly increased without affecting FBX, so that at pH 1.2 PXC was a co-former for FBX but at pH 6.8 and 7.4, FBX was a co-former for PXC. In vitro, the FBX–PXC co-crystal improved the dissolution time by up to 2.8-fold compared to pure PXC without affecting the dissolution of FBX. In addition, the FBX–PXC co-crystal had a stable crystal structure and altered mechanical properties such as an increase in the flow rate and the formation of capping and laminating at the time of compression, showing good compressibility [72].

NSAID, especially the anthranilic acid group, also can construct multi-APIs co-crystal with antibacterial drugs. This combination aimed to provide a unique combination drug or pain relief therapy and prevent postoperative infections. Hence, it minimizes or even replaces the use of opioids, reduces adverse effects, and improves the physicochemical properties of each API [30]. For example, niflumic acid (NFA)–caprolactam (CPR) (1:1) is an example of an anthranilic acid class of NSAID that selectively inhibits COX-2 and is widely used in patients with rheumatoid arthritis. The co-formers used to form NFA co-crystals are CPR and 2-hydroxy pyridine (2HP). An analysis of the crystal structure showed the type of crystals that form between the salt and the co-crystal together by bonding through NH···O and OH···O hydrogen bonds, which form dimers, and CH···F and CH···O. The NFA–CPR co-crystal has a low melting point of 83 °C. In contrast, the NFA-2HP co-crystal has a melting point of 135 °C [69].

Bhattacharya et al. performed a screening of the hydrate salts and multi-API co-crystals of anthranilic acid with an antibacterial. There are two co-crystals from APIs, namely flufenamic acid (FFA) with sulfamethazine (SFZ) and niflumic acid (NFA) and SFZ. In the crystal structure, hydrogen bonds are formed between the carboxylic acid groups of NFA/FFA with the sulfonamide (NH) groups and N atoms from the pyrimidine ring on the SFZ to form synthon III. Then, each dimer unit is connected by four pairs of identical NH···O hydrogen bonds to form synthon IV. This structure is stabilized by CH···interactions and π···π interactions [30].

Machado et al. conducted a study on an NSAID co-crystal formed from propionic acid with levetiracetam (LEV), which was expected to treat epilepsy accompanied by inflammation. LEV is an etiracetam enantiomer and acts as an oral anti-epilepsy drug classified as BCS class I (good stability and permeability), so it is expected to improve the physicochemical properties of the NSAID. The eutectic mixture and co-crystal dissolution test with aryl propionic acid (IBU, naproxen (NPX), Flurbiprofen), and LEV showed an increased dissolution rate compared to the pure NSAID. LEV + (S)-IBU crystals were stable in accelerated stability tests over six months. However, the eutectic mixture of propionic acid and LEV melted at temperatures below that of the pure NSAID. This suggests that the crystalline form of propionic acid and LEV can also increase propionic acid derived NSAID pharmacokinetic parameters [61].

Surov et al. conducted a study on the formation of co-crystal diclofenac (DFA), diflunisal (DIF) with theophylline (THP), as well as a DFA–THP co-crystal with a DIF-THP co-crystal; the co-crystal synthesis was carried out by grinding with the addition of mixed solvent droplets (acetonitrile, methanol, and water). The synthon that formed as a hetero synthon connected by O···HN involves carboxylic acid from API and an unsaturated N atom from the imidazole ring on theophylline. The DFA–THP co-crystal provides a lower energy grid than DIF–THP because the packing consists only of dispersion energy. Co-crystal DIF–THP had a lower melting point than pure DIF, causing the co-crystal to be less stable. The small co-crystal enthalpy indicated that the energy in the hydrogen bond was proportional to the parent compound. The packaging was strengthened by the weak interactions of van der Waals forces. Based on the intrinsic dissolution results, co-crystallization increased the solubility of DFA by up to 1.3 times. In contrast, in DIF, the solubility was comparable to that of pure DIF. In this study, DIF–THP dissolution illustrated the classic concept of “spring and parachute”. Apart from increasing the solubility of co-crystallization, it also increased the stability in different humidity [45].

Aitipamula et al. conducted a study of oxaprozin (OXP) co-crystal formation with the co-former 4,4 bipyridine (4.4 BP), 1,2 BPE and salts with piperazine, 2-amino-3-picoline, and anti-asthma drugs such as salbutamol [63]. OXP is included in the propionic acid group, which is used to relieve rheumatoid arthritis. It is thought to be able to inhibit urate reabsorption by selectively inhibiting glucuronidation [109]. There have been very few publications on the co-crystallization of OXP. OXP salt formation showed a decrease in intrinsic solubility and dissolution rate. Therefore, OXP co-crystal can be utilized in tablet formulation of extended-release SAL to overcome the short half-life problem and lower the frequency of SAL use. The co-crystals arrangement occurs at a stoichiometric ratio (1:0.5) with hydrogen interactions through the interactions CH···O and CH···π. The OXP-4,4 BP co-crystal had a monoclinic crystal form with one OXP molecule and half a molecule of 4.4 BP in asymmetrical units. In comparison, the OXP–1,2 BPE co-crystal produced a triclinic crystal form with one OXP molecule and half a molecule of 1,2 BPE. The co-crystal and the salt were relatively stable and not hygroscopic [63].

Drug–drug NSAID co-crystals can influence clinical practice, especially for pain management, gout therapy, and osteoarthritis. This phenomenon is related to the synergistic effect of analgesics without the potential to cause side effects, improving the physicochemical properties and clinical profile of drug release so that it is expected to reduce dosages, patient complaints related to drug use, and economic burden of treatment [110,111]. In addition, based on the literature description of NSAID co-crystal drugs, the combination of drugs with the same BCS class can improve drugs’ stability and mechanical properties, such as the flow rate and compressibility.

## 4. Challenges in NSAID Co-Crystal Development

The development of NSAID co-crystals is a complicated and lengthy process involving prediction, screening, synthesis, characterization, pre-formulation, and studies of pharmacokinetic profiles, including adsorption, distribution, metabolism, and excretion, followed by formulation, process development, preparation, declaration of an Investigational New Drug, and clinical trials [112]. The number of stages that must be passed certainly causes many significant challenges in the development of co-crystals.

### 4.1. Co-Former Selection

Choosing a co-former compatible with API is one of the challenges of co-crystal formation. Until now, the selection of co-formers was done by filtering the co-crystal by “tackles.” In this approach, crystals with the best physicochemical and pharmacological properties are selected, while the selection of co-formers is performed directly by trial and error. An alternative method that is often used is the supramolecular synthons approach, where the priority of the co-former is selected based on data analysis from the CSD. In determining co-formers, several parameters should be considered. The strength of the hydrogen bonds and Hansen’s solubility parameter are used to predict the theoretical solubility of drugs and co-formers. These are based on calculating partial solubility parameters with the Van Krevelen-Hoftyzer, Bagley, and Greenhalgh approach [7].

Cheney et al. conducted a study regarding the selection of co-formers in meloxicam (MLX) co-crystal formation to increase solubility and pharmacokinetics [74]. In this study, the co-former selection was carried out using the supramolecular synthons approach by analyzing the CSD to ensure the reliability of hetero-synthons or homo-synthon supramolecular formation between azole groups in MLX with carboxylic acids, primary amides, or alcohol. In the shape of the supramolecules, only hydrogen bonding interactions were considered. Based on the CSD search of 450 hits containing azole and a carboxylic acid, only 102 entries formed hetero synthon supramolecules with carboxylic acids. These results indicate that hetero-synthon supramolecules are predominantly formed, compared to homo-synthons. This result was also shown for azole–carboxylic and azole–alcohol consistently. The exception was primary amides, which contain more homo-synthon supramolecules. From these results, aspirin (ASP), which is an aromatic carboxylic acid, was selected as a co-former in co-crystal formation. However, based on the FDA approval base document, oral administration of MLX with ASP at a 1000 mg/day dose is not recommended because it can increase the AUC and Cmax of MLX, although until now, there have been no reported side effects with its use [7,74]. Furthermore, in selecting the co-former, the safety of the co-former is the most crucial factor. The co-former established in the co-crystal formation must be safe or non-toxic in the amount required as the therapeutic drug dose. Most co-crystal development is done using co-formers that have been registered as chemical additives deemed safe for human consumption, known as GRAS. This list includes various chemicals, including aldehydes, alcohols, carboxylic acids, amides, and sweeteners. Therefore, the structural diversity and the physicochemical properties of the substances in the GRAS list provide an additional means of selecting co-formers [30].

### 4.2. Solubility

Solubility enhancement is the main background of NSAID co-crystal development. However, some co-crystals showed no solubility improvement, i.e., niflumic acid co-crystals with sulfamethazine (SFZ) [30]. Next, ethenzamide (ET) co-crystallization with nutraceutical ingredients from hydroxybenzoic acid derivatives, namely sinapic acid (SNP), an antioxidant, antimicrobial, anti-inflammatory, and anti-cancer agent, led to lower solubility than single ET due to the poor solubility SNP. This finding indicates that the solubility of the co-former greatly influences solubility [42].

The solubility of some NSAID co-crystals depends on environmental conditions, such as pH and temperature. It was found that ET was soluble at an acidic pH of 1.2 due to chloride salt formation from protonated ET to form strong hydrogen bonds with an increase in dissolved molecules. At the same time, co-crystallization of this NSAID with 3,5 dihydroxybenzoic acid (DHBA) showed higher solubility at pH 7.4 than at pH 1.2. This shows that pH affects solubility. In addition, the conformation of a drug during the packing of the co-crystal lattice with co-former can cause molecular planarity, which leads to an increase in solubility [113]. As a ratio of the total concentration of the co-former to the whole drug in equilibrium, the crystal eutectic constant can predict the solubility at different pH [114].

**Figure 11:**
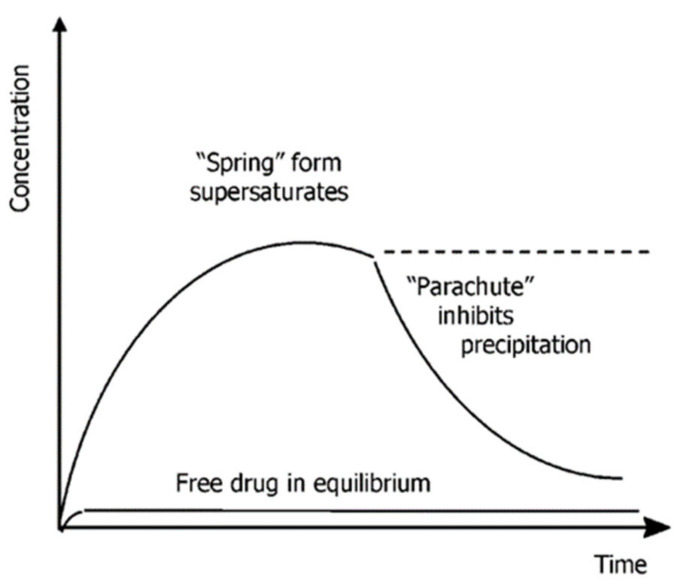
The spring and parachute approach to drug saturation in solution. Adapted with permission from ref. [115]. Copyright 2007 Elsevier.

Thermodynamic forms of high-energy drugs such as ionic salts and drug co-crystals with a highly soluble co-former provide the driving force to achieve drug supersaturation, called the “spring”. Unfortunately, this phenomenon carries a high risk of accelerating precipitation, causing worse solubility than before. Therefore, a combination of excipients that can inhibit or slow down the precipitation or formation of “parachute” crystals is required (Figure 11). This approach has been applied by Guzman et al. to increase the oral absorption of celecoxib (CEL) salt co-crystal in solid preparations [115]. In this study, they conducted experiments with several excipients in the form of a surfactant mixture to obtain a parachute effect, namely the parameters of deposition time and critical micelle concentration (CMC). The deposition time was the longest, with the lowest CMC concentration. Moreover, the excipient must also be able to increase a saturated concentration of CEL in the solution. A study was carried out in vivo using beagles to determine the drug formulation with the best pharmacokinetics compared to marketed CEL, namely Celebrex. This study found that the Pluronic F127 excipient showed the best parachute effect with the inhibition of precipitation at 37 °C and a CMC concentration of 0.07 mg/mL and slowed down the dissolution or change of the CEL salt co-crystal into acidic CEL crystals. Pluronic F127 can prevent the direct wetting of CEL solids and form cohesive clots related to its flower-like form. Based on in vivo and in vitro testing results, CEL co-crystals formulation consists of vitamin E tocopherol polyethylene glycol succinate, hydroxypropyl cellulose, and Pluronic F127 and provided 100% bioavailability with faster absorption compared to Celebrex. A linear increase in the AUC with dose indicated that the formulation had a quicker onset and a lower dose. This formulation has also been tested for stability for three years. In addition, the optimal arrangement of CEL co-crystal with appropriate excipient help can increase the drug’s solubility [115].

Spring and parachute effects also occur in INC co-crystals with SAC as the co-former. The co-crystal induces rapid dissolution followed by sudden drug precipitation within 60 min. This phenomenon is certainly a concern in NSAID co-crystals [47]. Using appropriate excipients to inhibit co-crystal precipitation is one way of dealing with this phenomenon [112]. In addition, the formation of a co-crystal significantly affects the solubility in the construction of a supramolecular matter where the absence of a synthon with few hydrogen bonds can cause a decrease in solubility. A study of the co-crystals of meloxicam (MLX) with glutaric acid (GLU), MLX with l-malic acid, and MLX with fumaric acid showed decrease in solubility, especially for MLX–GLU co-crystal, i.e., 14% lower than that of pure MLX. In this study, the high solubility of the co-former did not translate into increased solubility of the co-crystal. The absence of NH···O = S interactions between MLX can increase solubility, but this interaction was not correlated with equilibrium Cmax changes. The poor correlation between the melting point analysis and Cmax shows that the co-crystal lattice’s supramolecular arrangement was inconsistent. Intermolecular interactions between MLX molecules linked in a crystal lattice play only a limited role in crystal dissolution thermodynamics [75].

### 4.3. Permeability

A drug’s ability to penetrate biological membranes is crucial to increasing absorption and distribution to achieve its therapeutic effect. This factor is closely related to the bioavailability of a drug [112], so that decreased permeability is a problem in the formation of NSAID co-crystals for both oral and transdermal/topical preparations. The development of NSAID co-crystals for topical preparations requires very high permeability and suitable dosage formulations. The permeability will be strongly influenced by the viscosity of the pharmaceutical preparation, as well as its pH and lipophilic/hydrophilic properties. For example, MLX–salicylic acid (SA) co-crystal in a gel was administered transdermally and showed a significant decrease in permeability, i.e., up to 5.2-fold lower than the co-crystal suspension. The formation of supersaturation due to a reduction in pH can prevent the ionization of acidic medicinal compounds, thereby increasing free drugs in the medium and increasing the permeation rate [73].

Permeability is related to lipophilic and hydrophilic properties, which depend on the density of hydrogen bonds formed on the carboxylate and hydroxy groups in the co-crystal. An increase in hydrogen bonding will increase solubility; this was observed as a rise in the ET and 3.5 DHBA co-crystal permeation rate [40]. In the other cases, γ-indomethacin-2-hydroxy-4-methylpyridine and INC–SAC co-crystals increased permeability by two-fold compared to that of ordinary INC. The two co-crystals decreased the transepithelial electrical resistance value as a function of cell membrane integrity, i.e., 8.5-fold. Loss of the cell membrane monolayer (NCM460) as a barrier to drug absorption in the body can increase the drug’s solubility and bioavailability and speed up the time to reach the therapeutic dose of INC [15]. Apart from INC, some co-crystal NSAID drugs have been reported to increase permeability, such as the ethenzamide–saccharine (ET-SAC) co-crystal [113].

### 4.4. Intrinsic Dissolution Rate (IDR)

IDR in an aqueous medium is a crucial parameter in determining the solubility of a co-crystal. IDR is the dissolution rate of a pure substance under constant temperature, pH, and surface area conditions. This parameter provides a more significant correlation with in vivo dynamic dissolution than the solubility test. IDR is a tool for evaluating drug solubility in the BCS [116]. IDR is described as the cumulative amount of dissolved per unit surface of the drug preparation plotted against time in units (unit: mg/cm^2^/min). For example, co-crystallization of IBU with NIC could increase the dissolution rate by up to 2.5 times that of the single IBU [59]. The tramadol–celecoxib co-crystal (CTC) also increased dissolution rate three times more than CEL, which can accelerate absorption and increase bioavailability. The maximum concentration of CEL in CTC increased, compared to CEL alone [79]. Different co-crystal preparation methods result in various particle sizes and shape distributions, affecting the IDR. Based on previous research, co-crystallization between PCM and caffeine (CAF) produces co-crystals A, B, C, D, E, which showed increased IDR values 1.72, 1.88, 2.42, 2.19, and 2.84 times higher than that of IDR from PCM (5.06 mg/cm/min) [82]. The IDR can be comparable to or even less than the dissolution rate of a single NSAID drug, such as the IBU-NIC and flurbiprofen (FLU)–NIC co-crystals, which prolonged IDR around eight times lower than IBU and five times lower than FLU [117].

### 4.5. PH Microenvironment

**Figure 12:**
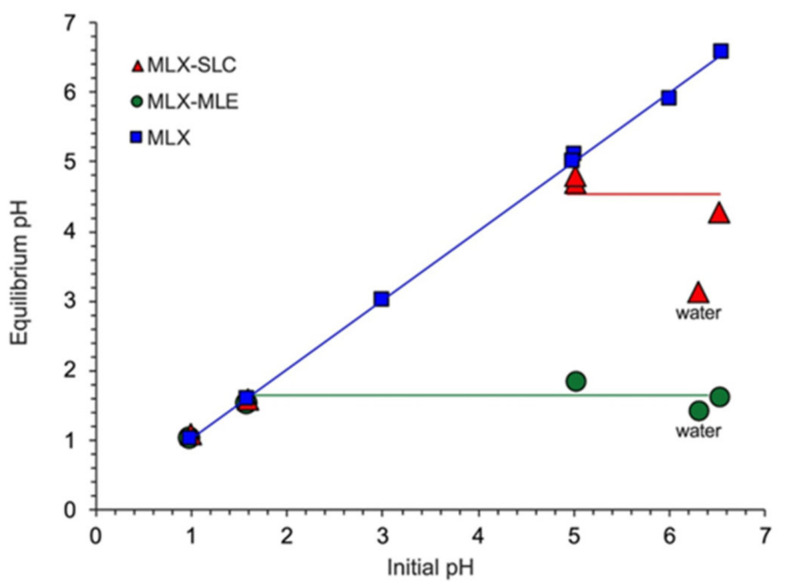
The initial pH value and equilibrium of the solubility of the drug at the eutectic point. Reprinted with permission from ref. [118]. Copyright 2020 Elsevier.

The pH microenvironment plays a vital role in the solubility and dissolution of the co-crystal. This is shown by the initial pH (pHint) relationship representing the bulk pH, with equilibrium pH representing the microenvironment pH (Figure 12). In a co-crystal, pHint will reach equilibrium at the eutectic time, in which the solution is highly saturated with the co-crystal and drug. In contrast to drugs, where pHint = bulk pH, for co-crystals pHint <bulk pH. This statement shows that the co-crystal is highly dependent on pHint, especially for acidic co-formers, lowering the interface pH. Machado et al. demonstrated that the formation of MLX–MLA co-crystals and MLX–SA could reduce the microenvironment pH to 1.6 and 4.5, respectively [118]. This decrease occurs due to the nature of co-crystal ionization, which causes changes in solubility and dissolution in the MLX–SA co-crystal. In this case, reducing the microenvironment pH of the MLX–MLA co-crystal can reduce the solubility dependence of the MLX–SA co-crystal on pH [118]. The effect of pH on solubility also occurred in PXC–SAT co-crystal. PXC formed a sigmoid curve, showed that solubility of PXC did not change until pH 5, then increased rapidly due to the ionization process at alkaline pH caused by the weak acid nature of PXC [33]. In another publication, LNX solubility concerning pKa increased at pH 4.5, whereas the LNX co-crystal increased at pH 7.4, indicating pH-dependent ionization [109]. Hereafter, it is essential to know the pKa of the drugs and co-formers in a co-crystal to predict the effect of the microenvironment pH [19].

### 4.6. Stability

Co-crystal stability is a parameter that determines the potential for further development of pharmaceutical products. NSAID co-crystal development will significantly depend on chemical and physical stability. Hereafter, stability properties are undoubtedly the big challenge in co-crystallization. Practically, stability testing is carried out on various aspects, namely moisture stress, thermal stress, and chemical stability [119].

#### 4.6.1. Moisture Stability

The stability test results at a temperature of 40 °C and a humidity of 75% RH, 82% RH, and 96% RH for two weeks showed no change in the mefenamic acid-N-methyl-d-glucamine (MFA–MG) co-crystal, indicating that the co-crystal was stable in high humidity [66]. This test provides information that the water content caused molecular damage to determine co-crystal shelf life and storage [119]. Moisture can cause co-crystal distortion, conversion to a hydrate form, or dissociation [19]. Different methods conferred differences in stability, such as in the moisture stability study of the PCM co-crystal with the co-former OXA formed by the grinding method and the PCM–MLA co-crystal formed by solvent evaporation. CM–MLA co-crystal was more stable than PCM–OXA, because, under wet conditions OXA tended to be converted to the dihydrate form. This study also concluded that co-crystal stability is related to solvent polarity, whereas PCM–OXA is more stable in aprotic solvents [29]. Another study showed that the diclofenac–proline co-crystal (DFA–PRO) could also dissociate in the humidity levels above 80–90%. It was represented by low intensity on the diffractogram and diffraction peaks and a high-intensity PRO signal after 24 h of storage at 80% RH and 12 h of storage at 90% RH. Moreover, the results showed that the co-crystal was stable at 75% RH [48]. This data suggested DFA co-crystal stability was influenced by humidity. In this case, the unstable co-crystal at high humidity must be modified and retested into other products [19].

#### 4.6.2. Chemical Stability

Accelerated stability testing was carried out under a temperature of 40 °C and 75% humidity. Chemical stability is related to co-crystal component degradation due to an API incompatibility with the co-former. The PXC–sodium acetate (SAT) co-crystal in tablet form showed no changes in color, odor, hardness, brittleness, drug content, disintegration time, or percentage dissolution after being stored at 40 °C/75% RH for three months [33].

#### 4.6.3. Thermal Stability

This stability test must be carried out at a high temperature and pressure to assess co-crystal stability against temperature increases [119]. In several studies, stability testing was carried out with differential scanning calorimetry (DSC) to observe co-crystal thermal stability. For example, Oswald et al. prepared PCM co-crystals with several co-formers, i.e., 4-dioxane, *N*-methyl morpholine, morpholine, N, *N*-dimethyl piperazine, piperazine, and 4,4-bipyridine. Of the five co-crystals formed, only PCM-4,4 bipyridine did not decompose at a high temperature due to the high boiling point of 4,4-bipyridine (578 K). Thus, the boiling point of each co-former would significantly influence the co-crystal’s thermal stability [120]. 

There are unexpected occurrences when co-crystals have a lower melting point than the parent component. This case occurred in ketoprofen (KET) and MAL co-crystals in the ratios 1:1, 1:2, and 2:1. Based on the KET–MAL co-crystal DSC analysis, the endothermic peak showed the melting point of each co-crystal proportion at 86.6, 79.5, and 86.2 °C, while the KET melting point was 96.1 °C; MAL had two endothermic peaks at 104.2 and 135.6 °C [121]. The instability due to co-crystallization was also shown by the CEL–NIC system, in which the co-crystals dissociate at 25 °C into a more stable single form [81]. Lower endothermic temperature peak also presented in the ET–SAC co-crystal with Tonset = 123.68 °C [41].

**Figure 13:**
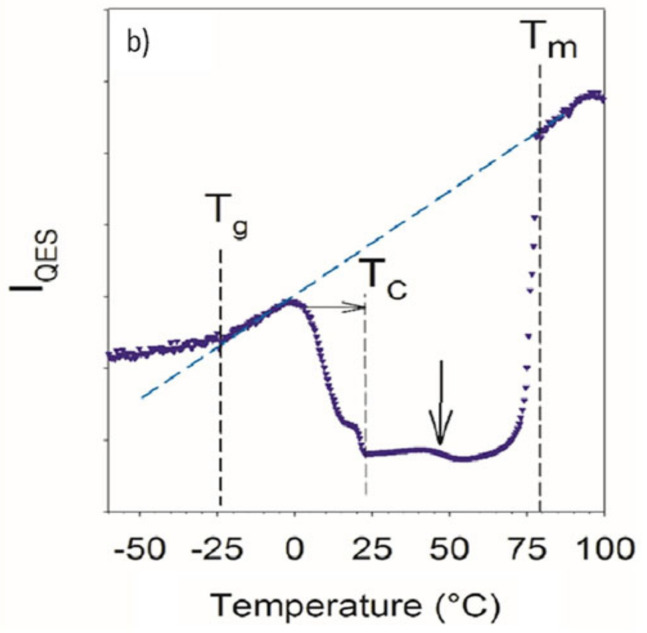
Transforming shape A into B (vertical arrow) and crystallization (horizontal arrow) of IBU-NIC (ibuprofen-nicotinamide) co-crystal. Adapted with permission from ref. [93]. Copyright 2020 Elsevier, 2020.

In addition to DSC and PXRD analysis, Guerain et al. also used low-frequency Raman spectroscopy to investigate findings that could not be explained by PXRD, especially for molecular systems in low-electron atoms. This test was carried out under thermal conditions similar to those used in DSC analysis. It produced a shallow frequency spectrum (<50 cm^−1^), indicating the status of the S-IBU–NIC co-crystal, which showed limited physical stability over a relatively narrow temperature range. Figure 13 shows the occurrence of a glass transition state followed by a recrystallization process characterized by a curve reduction and reveals a transformation at 50 °C that was not detected in the DSC analysis. This result is because the low-energy phase transition is difficult for DSC to observe, which has a relatively weak scan rate (0.5 °C/min). The recrystallization of S-IBU–NIC occurred in two successive phase transitions: form A underwent a polymorph transformation into a unique and stable form B. This fact indicates the difficulty of achieving a steady state by the recrystallization method, so it is crucial to consider the preparation method for obtaining stable crystals [93].

### 4.7. Mechanical Properties of NSAID API

External forces are essential in drug development because the drug undergoes grinding, filling, molding, and compacting of the powder, which can cause physical deformation. Therefore, the co-crystallization technique can be used for alternative crystal packaging and improving APIs’ mechanical properties [14]. The mechanical properties can be assessed by the nanoindentation, elasticity (E), and hardness (H) of the measured material load transfer. E and H are measures of the resistance of the material to plastic elasticity and deformation. High E and H values indicate that the material is resistant to deformation and is brittle. However, based on several studies, co-crystallization does not continually improve mechanical properties, such as the co-crystal ibuprofen–lysine (IL) with polyvinylpyrrolidone K25 (PVP K25) and polyvinylpyrrolidone K30 (PVP K30) as co-formers, which have compressibility properties comparable to IL [122]. 

Wicaksono et al. synthesized KET–MLA co-crystals by forming a C=O ketone group interaction between KET and MLA. Based on hot stage microscopic analysis and SEM, the KET–MLA co-crystal produced a needle-shaped, bigger particle size than KET, with a multi-shaped rough surface on the co-crystal [121]. Additionally, Karki et al. applied a co-crystallization technique to increase PCM tabletability with several co-formers, namely OXA, THP, naphthalene, and phenazine. The PCM co-crystal with the lowest shear stress value was obtained with low tensile strength based on testing [7]. Chattoraj et al. showed that the co-crystallization of PXC–SAC decreased plasticity and increased elasticity, which significantly reduced the compacting properties of API and its co-former, thought to be caused by suboptimal packaging of the co-crystal [123]. 

A naproxen–nicotinamide co-crystal (NPX–NIC), produced with low tensile strength (<2 MPa), caused lamination and clumping in tablets [124]. Conversely, several studies have shown that co-crystallization can improve the mechanical properties of NSAID co-crystals such as PCM–CAF. The liquid-assisted grinding (LAG) method is the best method to modulate the physical, mechanical, and pharmacokinetic properties, which resulted in a more delicate PCM–CAF co-crystal powder with a high compressibility index (31.12%) and high tablet hardness. Furthermore, the ratio of the API to the co-former can affect the mechanical properties, as shown in a PCM–CAF 1:1 co-crystal (A, B, and C) and a PCM–CAF 2:1 co-crystal (D and E) prepared by the solvent evaporation method with different solvents. The Heckel plot of the co-crystal showed increased compacting due to high plasticity. The plasticity was indicated by the ratio of the mean yield pressure, which is the stress of particle deformation that occurs during compression. The plasticity of the crystals showed different values due to the differences in the molecular packaging features of each co-crystal. Co-crystals A, B, and C had better plastic properties and higher tensile strength than co-crystals D and E [82]. Improved mechanical properties also occurred in flufenamic acid (FFA)–NIC co-crystals, which showed better tablet properties than FFA [68].

### 4.8. Polymorphism

Polymorphs of drug substances have different physical and chemical properties, affecting drug products’ safety, quality, and effectiveness [125]. Co-crystallization can exhibit polymorphisms in various crystal structures. Conformation changes in co-crystallization to form efficient hydrogen bonds can lead to polymorphism, i.e., by rotating the arrangement of C–C–C–O bonds. For example, there are three polymorphic forms of DIC co-crystallization with pyridine-based co-formers. The flexibility of DIC in salts and co-crystals allows the molecules to have different solid-state conformations [25].

Several studies have reported co-crystal polymorphs, such as an experiment conducted by Child et al. [126]. The co-crystallization of PXC/4-hydroxybenzoate (1:1) produced two polymorphs. Polymorph I had an un-ionized PXC tautomer with the co-former and polymorph II with a zwitterionic tautomer with the co-former. The co-crystals formed in this study included 2:1 piroxicam/succinic acid, 1:1 piroxicam/1-hydroxy-2-naphthoic acid and 1:1 piroxicam/caprylic acid, 1:1 piroxicam/malonic acid, 4:1 piroxicam/fumaric acid, and 1:1 piroxicam/benzoic acid [126]. Hereafter, the formation of polymorphs is a considerable challenge because it can transform during the pharmaceutical preparations and changes the drug’s performance.

**Figure 14:**
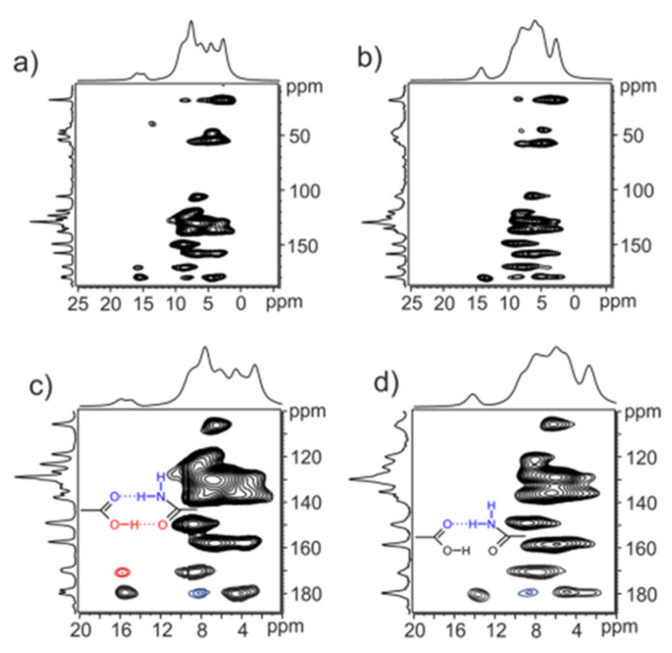
Inv-HETCOR results at 42 kHz for (**a**,**c**) α and (**b**,**d**) β type NPX-PA co-crystals. Reprinted with permission from ref [36]. Copyright 2016 American Chemical Society.

Humidity and mechanical stress are the main factors leading to the formation of polymorphs, i.e., NPX-picolinamide (PA) produced two polymorphs, namely α and β. The α polymorph was unstable and tended to undergo a phase transition to the β form at 95 °C. Identification of the polymorph by very fast mass nuclear magnetic resonance, an advancement in MAS NMR technology, showed that the two polymorphs had different structural properties, based on the results of the heteronuclear correlation (HETCOR) test. It concluded that the α and β co-crystals had different hydrogen patterns, where the α co-crystal had a type I synthon (NPX as the acceptor, the amide group of PA as the donor) and the β co-crystal had a type II synthon due to the rearrangement of the structure during thermal processing below the melting point (Figure 14) [36].

The co-crystallization of ET and resorsilic acid, namely 2,4-DHBA, produced three polymorphs with different conformations on the amide groups, which provided other physicochemical properties. Polymorph I from ET and 2,4-DHBA (1:1) was yielded from a solution without formic acid, while the formic acid addition produced polymorph II. Lastly, polymorph III was formed from a mixture of ET and 2,4-DHBA (2:1) with formic acid in the mixture solution. Polymorphs I and II had good stability, non-hygroscopic character, and higher solubility at pH 7.4. In this study, the solubility increase was related to the decrease in melting point, reflecting lower lattice energy. Besides, the co-crystal polymorphs had a smaller particle size than ET, so the solubility was better than ET [40].

### 4.9. Development of NSAID Formulations

There are three main stages in producing a co-crystal product: formulation, process, and packaging to maintain co-crystal stability. These steps create many new challenges for different environments. Hence, it is first necessary to perform a preformulation. An essential preformulation step is to ensure that the excipient is compatible with the co-crystal. Incompatibility of the excipient against the co-crystal, especially its co-former, can lead to co-crystal destruction and hydrolysis, which cause physical instability and chemical incompatibility [19]. Crucially, the addition of a suitable excipient can increase solubility, stability, dissolution, and oral absorption. Remenar et al. conducted a study to expand PVP-K30 and sodium dodecyl sulfate (SDS) excipients for CEL–NIC co-crystal formulas. The results showed that low surfactant concentrations converted large aggregates of CEL-III, which reduced the dissolution rate. However, the addition of 1–10% solid SDS and PVP converted the crystalline form into amorphous and crystalline micron forms (CEL-IV), which increased the bioavailability up to four times more than CEL in Celebrex. All co-crystals dissolved in 2 min in 1% SDS solution, showing that this co-crystal formulation has the potential to overcome the “spring and parachute” effect [127]. Lactose monohydrate (LA), potato starch (PS), and potassium bromide (PR) are known to prevent changes in the polymorphisms of ET and gentisinic acid (ETGA) 2, which was less stable at high pressure compared to ETGA 1 in the tableting process indicated by the absence of peak shift and split in solid-state nuclear magnetic resonance (SSNMR) 13C and 15N and FTIR. ETGA 1 was produced by slow evaporation with acetonitrile as the solvent at room temperature, while ETGA 2 was made using a solvent mixture of toluene–acetonitrile (1:1). The dissolution test showed that almost all co-crystal formulas (ETGA 1 and ETGA 2) with excipients (LA, PS, and PR), except for ETGA with LA, had a faster and greater dissolution than ET [39].

Panzade et al. optimized piroxicam–sodium acetate (SAT) co-crystal formulation to obtain several orodispersible tablets. A preliminary study was carried out with a 32 factorial design to optimize the super-disintegrant (sodium croscarmellose) concentration and the binder (PVP K-30), with formulation factors for evaluation. Various pre-compression and post-compression parameters indicated that all formulated tablets had a uniform weight with acceptable weight variations and thicknesses. All formula hardness was 3.2–3.6 kg/cm^2^, and the friability was found between 0.72–0.86%, indicating that the tablets had good mechanical resistance. The API content in the oro-dispersible tablets was around 98.04–99.48%, which is within the acceptable limits. F1 was the optimal formula with a disintegration time of 29 ± 0.12 s, a wetting time of 21 ± 0.58 s, a maximum water absorption ratio of 97.65 ± 0.25%, and a maximum concentration of the tablet dissolution test of 93.69 ± 0.12%. Apart from that, F1 was also stable in accelerated stability testing. ANOVA supported these results on the variable disintegration and dissolution times of the super-disintegrant and binder used. The super-disintegrant and binder concentration optimization had a significant effect (*p* < 0.05) on increasing the disintegration and dissolution time [33].

Of the nine polymorphs of flufenamic acid (FFA), the most widely used in the formulation were flufenamic acid form 1 (FFA 1) and flufenamic acid form 3 (FFA 3). Guo et al. constructed co-crystal from FFA 1 with the co-formers NIC and THP. The polymers used were polyethylene glycol (PEG), PVP, and polyvinylpyrrolidone–vinyl acetate. The formation of co-crystals was confirmed by the presence of shifts and new peaks in the PXRD diffraction pattern and FTIR spectrum and the difference in melting points of the co-crystal compared to the parent compound. The solubility of the FFA–NIC co-crystal in ethanol:water (1:4) was higher than FFA and FFA–THP. Moreover, the presence of polymers in the solvent did not change the solubility properties. In residue testing, insoluble compounds showed that the co-crystal changed into FFA 3. DSC results depicted that FFA 3 melted at 123.1 °C and, following recrystallization into FFA, melted at 134.4 °C [67].

The development process requires identifying parameters and factors that can affect the formation of both co-crystal and pharmaceutical preparation products. During the co-crystal formation process, knowledge of the physical and chemical properties of the starting materials is undoubtedly needed [16], as shown in the development process for sodium naproxen–lactose-tetrahydrate (S-NPX–LT) co-crystals. During the heating process, the S-NPX–LT co-crystal lost water molecules at a temperature of 60–120 °C, so co-crystal transformation occurred into a co-amorphous system that could change back to the co-crystalline form at high humidity. This study showed that water molecules are critical to stabilizing the co-crystal packaging. This case illustrates the challenges in process development for co-crystallization [128].

## 5. Conclusions

Co-crystallization is a solid formation process in crystalline form, which is very promising for NSAID development due to its ability to improve physicochemical and mechanical properties. Safer and more effective API produced by co-crystallization could update old drugs and encourage the evergreening drug patents. Co-crystals have been successfully constructed from various NSAID drugs with GRAS co-former, with the different hydrogen bond synthon motifs. Practically, the solid-state-based technique is the most effective method for forming NSAID co-crystals. NSAID co-crystallization renewal continues by selecting a co-former based on the CSD database, optimizing the scale-up procedure, and improving physicochemical properties by co-crystal drug delivery modification. The NSAID co-crystals also have been shown to accelerate the onset of action, extend the drug’s duration of action, and decrease the tolerance doses. NSAID co-crystal development for medicinal products faces many challenges, mainly in selecting safe co-formers and unpredictable conditions. The “spring and parachute” phenomenon, Ostwald purification, the decreased solubility and dissolution rate due to the influence of environmental pH, co-crystalline transformation caused by temperature, humidity, chemical changes, polymorphism, and the in-compatible excipients in the formulation, may exist in co-crystal development. Detailed scientific understanding of the ingredients’ properties in the preformulation, co-crystallization techniques, supramolecular interactions formed, and their manifestations to the biopharmaceuticals profile will provide the opportunities for the new NSAID product development.

## Figures and Tables

**Table 1:** NSAID co-crystal development.

Group of NSAID	API-Coformer	Ratio	Preparation Methods	Advance	Refs
Salicylic acid derivatives	Ethenzamide-gentisinic acid	1:1	Solvent evaporation	Increased dissolution rate and is more stable at high pressures.	[39]
	Ethenzamide-2,4 dihydroxybenzoic acids	1:1	Neat grinding	Has good stability, non-hygroscopic, higher solubility.	[40]
	Ethenzamide-saccharine	1:1.1	Neat grinding	Significantly increases solubility and permeability.	[41]
	Ethenzamide-sinapic acid	1:1	Solvent evaporation	Has good thermal stability.	[42]
	Diflunisal-nicotinamide	2:1	Solution co-crystallization	Thermal stability, improvements to solubility and dissolution.	[43]
	Diflunisal-isonicotinamide	2:1	Solution co-crystallization		
	Diflunisal-pyrazine	2:1	Ball milling		[44]
	Diflunisal-theophylline	1:1	Solvent-drop grinding	Increased solubility up to 2.3-fold, indicated for asthma therapy.	[45]
Indole	etodolac-piperazine	2:1	Solvent-drop grinding	Sstable and increased solubility of todolace.	[46]
	Etodolac-isonicotinamide	1:1			
	Indomethacin-l-proline	1:1		Increased solubility, bioavailability, permeability up to 2-fold and improved pharmacokinetic profiles such as increasing cmax up to 1.6-fold in 1h, accelerating tmax, and extending the drug half-life.	[23]
	Indomethacin-saccharine	1:1	Refrigerant crystallization method without seeding	Increased stability, bioavailability, solubility and intrinsic dissolution rate up to 1.7-fold at pH 1.2.	[47]
	Indomethacin-2-hidroksi-4-metilpiridin	1:1	Slow solvent evaporation	Increased permeability up to 2-fold.	[15]
Heteroacil acetic acid	Diclofenac-l-proline	1:1	Liquid assisted grinding and neat grinding	Increased stability and solubility up to 7.69-fold.	[48,49]
	diclofenac-theophylline	3:1	Solvent-drop grinding	Increased dissolution rate up to 1.3-fold, indicated for asthma therapy.	[45]
	Diclofenac-ethyl diclofenac	1:1	Solvent evaporation	Increased chemical stability and inflamatory activity.	[50]
	Sodium-diclofenac-l-proline-tetrahydrate	1:1:1:4		Increased stability and solubility up to 3.1-fold.	[27]
	Sodium-diclofenac-l-proline-monohydrate	1:1:1:1		Increased dissolution rate and solubility up to 4.8-fold.	
	Potassium-diclofenac-l-proline-hydrate	1:1:1:4	Slow evaporation and aided by ultrasonic	Increased solubility and intrinsic dissolution rate salt potassium diclofenac up to 3.55-fold.	[51]
	Nano-diclofenac-l-proline	1:1	Top-down with the ng and bottom-up with microwaving assisted rapid evaporation	Increased solubility up to 2.64-fold and increased diffusion up to 2.06 fold that of the dfa-pro co-crystal.	[52]
	Aceclofenac-nicotinamide	1:1	Neat grinding	Reduced size, increased bioavailability up to 1.77-fold.	[31,53]
	Aceclofenac-gallic acid	1:1	Solvent evaporation	Increased bioavailability of co-crystal up to 1.73-fold.	[54]
	Aceclofenac-lysine	1:1	Solvent drop grinding	Higher melting point than parent drug, 201 °C	[55]
Arylpropionic acid	Ketoprofen-nicotinamide	2:1	Solvent evaporation	Increased solubility up to 1.3-fold.	[56]
	Naproxen-nicotinamide	2:1	Supercritical antisolvent	Size distribution ranging between 20 μm and 1 mm and flow ratio increased to 36 times.	[57]
	Naproxen-urea	1:3	Solvent evaporation	Increased dissolution rate up to 4.37-fold, has good powder flow properties and compressibility.	[58]
	Naproxen-thiourea	1:2	Solvent evaporation	Increased dissolution rate up to 4.5-fold.	
	Ibuprofen-nicotinamide	1:1	Solvent evaporation	Increased solubility up to 70-fold, dissolution rate up to 2.5-fold and improved the level of pain inhibition up to 2-fold.	[17,59]
	Flurbiprofen-benzamide	1:1	Liquid-assisted grinding	Increased solubility, intrinsic dissolution rate, and showed a swift and massive dissolution within the first 1h.	[60]
	Flurbiprofen-picolinamide	1:1	Solvent evaporation	Increased dissolution rate up to 4.37-fold, has good powder flow properties and compressibility.	
	Ibuprofen (R,S)-leverasetam	1:1	Green neat mechanochemical	Increased dissolution rate up to 2.5-fold, indicated to treat epilepsy accompanied by inflammation.	[61]
	Zaltoprofen-nicotinamide	1:1	Dry milling and solution-assisted grinding methods	Increased solubility up to 149-fold, dissolution up to 2.25-fold, thermal stability, and improved micromeritic properties, especially the flow rate of zaltoprofen.	[34,62]
	Oxaprozin-1,2-bis (4-pyridyl) ethane	1:0.5	Solvent evaporation	Stable and not hygroscopic.	[63]
Anthranilic acid (phenamic)	Mefenamic acid-nicotinamide	1:2	Melt crystallization	Increased solubility up to 1.6-fold.	[64]
		5:1	Gas anti-solvent	Increased the dissolution rate about 10–38-fold.	[65]
	Mefenamic acid-n-methyl-d-glucamine	1:1	Solvent evaporation	Increased dissolution up to 3.3-fold.	[66]
	Flufenamic acid-nicotinamide	1:1	Solvent evaporation	Increased solubility up to 1.1-fold and has good tabletability.	[67,68]
	Flufenamic acid-sulfamethazine	1:1	Liquid-assisted grinding	An increase in solubility of about 2.9–4-fold, indicated for pain relief therapy and preventive postoperative infections.	[30]
	Niflumic acid-caprolactam	1:1	Liquid-assisted grinding	Increased solubility up to 1.4-fold and dissolution rate up to 2-fold, indicated for pain relief therapy and preventive postoperative infections.	[69]
Enolic acid	Tenoxicam-resorcinol	1:1	Liquid-assisted grinding	Increased solubility up to 10-fold and intrinsic dissolution rates up to 8-fold.	[70]
	Piroxicam-sodium acetate	1:1	Dry grinding	Increased solubility up to 5-fold and dissolution up to 6.34-fold.	[33]
	Piroxicam-clonixin-ethyl acetate	1:1	Slurry methods	Improved stability at high humidity up to 95% RH. Synergistic therapeutic of anti-inflammatory.	[71]
	Piroxicam-febuxostat	1:1	Solvent evaporation	Increased stability, flow rate, compressibility, solubility febuxostat in pH medium 1.2–4.5 up to 2.5-fold, increased solubility piroxicam at pH 6.8 and 7.4, and improved dissolution rate piroxicam up to 2.8-fold in ph medium 1.2–4.5. indicated for gout therapy as uricosuric, anti-inflammatory, and analgesic agent.	[72]
	Meloxicam-salicylic acid	1:1	Reaction crystallization method	Increased solubility up to 5.7-fold and increased permeation rate up to 1.6-fold.	[73]
	Meloxicam-aspirin	2:1	Solvent evaporation	Increased solubility up to 44-fold in ph 7.4 medium, bioavailability up to 4.3-fold, accelerates the t max up to 4-fold (t max = 10 menit) and increases dose tolerance by up to 10-fold, indicated as a reliever of mild to moderate acute pain.	[74]
	Meloxicam-1-hydroxy-2-naphthoic acid	1:1	Solvent drop grinding	Increased dissolution rate up to 2.8-fold and drug absorption to 30.2 μg/mL within 21 min.	[75]
	Lornoxicam-sodium saccharin	1:1	Neat grinding method	Improved micromeritic properties, solubility, dissolution rate up to 2-fold and good stability in extreme temperatures.	[76]
	Lornoxicam-saccharin	1:1	Liquid-assisted grinding		[77]
	Lornoxicam-2,4 dihydroxy benzoic acid	1:1	Solvent drop grinding	Increased solubility up to 1.76-fold in pH 7.4 buffer and dissolution rate up to 1.52-fold at 10 min.	[78]
	Lornoxicam-l-catechol	1:1		Increased solubility up to 2-fold in pH 7.4 buffer and dissolution rate up to 1.25-fold at 10 min.	
COX-2 selective inhibitors	Celecoxib-tramadol HCl	1:1	Grinding co-crystallization method	Improved dissolution rate up to 3-fold, bioavailability, and reduce tramadol dose in the cocrystal, indicated for moderate to severe acute pain.	[79,80]
	Celecoxib-nicotinamide	1:1	Solvent evaporation	Increased stability of celecoxib.	[81]
Para-aminophenol derivatives	Paracetamol-caffeine	1:1	Liquid-assisted grinding	Increased relative oral bioavailability and improved intrinsic dissolution up to 2.84-fold	[29]
	Paracetamol-oxalate	2:1	Grinding process		
		1:1			
	Paracetamol-caffeine	1:1	Solvent evaporation	Improved stability thermal of drug.	[82]
	Paracetamol-indomethacin	1:1	Solvent evaporation method	Improved physicochemical properties.	[83]
	Paracetamol-mefenamic acid	1:1			

## Abbreviations

1,2 BPE	1,2-bis(4-pyridyl) ethane
2-apy	2-aminopyridine
4,4 BP	bipyridine
ACF	aceclofenac
API	active pharmaceutical ingredient
ASP	aspirin
AUC	area under curve
BCS	biopharmaceutics classification system
CAF	caffeine
CD.MOF	cyclodextrin metal−organic framework
CEL	celecoxib
Cmax	concentration maximum
CMC	critical micelle concentration
CMC-S	carboxymethylcellulose sodium
CNX	clonixin
COC-LG	co-crystal—loaded with liposome gel
CPR	caprolactam
CSD	Cambridge structural database
COX	cyclooxygenase
CTC	co-crystal tramadol celecoxib
DFA	diclofenac
DFT	density functional theory
DHBA	dihydroxybenzoic acid
DIF	diflunisal
DSC	differential scanning calorimetry
DTA	differential thermal analysis
EA	ethyl acetate
ED	ethyl diclofenac
ET	ethenzamide
ETGA	ethenzamide-genticinic acid
FBX	febuxostat
FDA	food drug administration
FFA	flufenamic acid
FLU	flurbiprofen
FTIR	Fourier transform infrared
GLA	gallic acid
GLU	glutaric acid
GRAS	generally recognized as safe
HETCOR	heteronuclear correlation
HPMC	hydroxy-propyl-methylcellulose
IBU	ibuprofen
IDR	intrinsic dissolution rate
IL	ibuprofen—lysine
INC	indomethacin
KET	ketoprofen
KET-NIC	ketoprofen-nicotinamide
LA	lactose
LEV	leveracetam
LAG	liquid assisted grinding
LNX	lornoxicam
LYS	lysine
MCR-ALS	multivariate curve resolution alternating least squares
MFA	mefenamic acid
MG	N-methyl-d-glucamine
MSP	mesopore
MLX	meloxicam
MLA	maleic acid
NDPC	nano-diclofenac-proline-co-crystal
NFA	nifluminic acid
NG	neat grinding
NIC	nicotinamide
NMR	nuclear magnetic resonance
NPX	naproxen
NSAID	non-steroidal anti-inflammatory drugs
OXA	oxalic acid
OXP	oxaprozin
PA	picolinamide
PXRD	powder x-ray diffraction
PCM	paracetamol
PVP	poly-vinyl-pyhrolidone
PXC	piroxicam
PR	potassium bromide
PRO	proline
PS	potato starch
SA	salicylic acid
SAC	saccharine
SAS	supercritical anti-solvent
SAT	sodium acetate
SDH	sodium diclofenac hydrate
SDPM	sodium diclofenac l-proline monohydrate
SDPT	sodium diclofenac l-proline tetrahydrate
SDS	sodium dodecyl-sulfate
SFZ	sulfamethazine
SNP	sinapic acid
S-NPX-LT	sodium naproxen-lactose-tetrahydrate
SS	sodium saccharine
SSG	sodium starch glycolate
SCXRD	single-crystal x-ray diffraction
TCA	d-tartaric acid
TGA	thermogravimetric analysis
TLC	thin-layer chromatography
THP	theophylline
VF MAS NMR	very fast mas nuclear magnetic resonance
XO	xanthine oxidase
ZFN	zaltoprofen
